# Host genetic susceptibility underlying SARS-CoV-2-associated Multisystem Inflammatory Syndrome in Brazilian Children

**DOI:** 10.1186/s10020-022-00583-5

**Published:** 2022-12-12

**Authors:** Cíntia Barros Santos-Rebouças, Rafael Mina Piergiorge, Cristina dos Santos Ferreira, Raquel de Seixas Zeitel, Alexandra Lehmkuhl Gerber, Marta Cristine Felix Rodrigues, Ana Paula de Campos Guimarães, Rodrigo Moulin Silva, Adriana Rodrigues Fonseca, Rangel Celso Souza, Ana Tereza Antunes Monteiro de Souza, Átila Duque Rossi, Luís Cristóvão de Moraes Sobrino Porto, Cynthia Chester Cardoso, Ana Tereza Ribeiro de Vasconcelos

**Affiliations:** 1 Departamento de Genética, Instituto de Biologia Roberto Alcantara Gomes, Universidade do Estado do Rio de Janeiro, Rio de Janeiro, Brazil; 2 Laboratório de Bioinformática - LABINFO, Laboratório Nacional de Computação Científica, LNCC/MCTIC, Getúlio Vargas, Av., 333, Quitandinha, Zip Code: 25651‑075 Petrópolis, Rio de Janeiro, Brazil; 3 UTI Pediátrica, Hospital Universitário Pedro Ernesto, Universidade do Estado do Rio de Janeiro, Rio de Janeiro, Brazil; 4 Serviço de Reumatologia Pediátrica, Instituto de Puericultura e Pediatria Martagão Gesteira - IPPMG, Universidade Federal do Rio de Janeiro, Rio de Janeiro, Brazil; 5 Laboratório de Virologia Molecular, Universidade Federal do Rio de Janeiro, Rio de Janeiro, Brazil; 6 Laboratório de Histocompatibilidade e Criopreservação, Universidade do Estado do Rio de Janeiro, Rio de Janeiro, Brazil

**Keywords:** Multisystem Inflammatory Syndrome in Children, SARS-CoV-2, COVID-19 complications, Host genetics, Admixed population

## Abstract

**Background:**

Multisystem Inflammatory Syndrome in Children (MIS-C) is a life-threatening complication of severe acute respiratory syndrome coronavirus 2 (SARS-CoV-2) infection, which manifests as a hyper inflammatory process with multiorgan involvement in predominantly healthy children in the weeks following mild or asymptomatic coronavirus disease 2019 (COVID-19). However, host monogenic predisposing factors to MIS-C remain elusive.

**Methods:**

Herein, we used whole exome sequencing (WES) on 16 MIS-C Brazilian patients to identify single nucleotide/InDels variants as predisposition factors associated with MIS-C.

**Results:**

We identified ten very rare variants in eight genes (*FREM1*, *MPO*, *POLG*, *C6*, *C9*, *ABCA4*, *ABCC6*, and *BSCL2*) as the most promising candidates to be related to a higher risk of MIS-C development. These variants may propitiate a less effective immune response to infection or trigger the inflammatory response or yet a delayed hyperimmune response to SARS-CoV-2. Protein–Protein Interactions (PPIs) among the products of the mutated genes revealed an integrated network, enriched for immune and inflammatory response mechanisms with some of the direct partners representing gene products previously associated with MIS-C and Kawasaki disease (KD). In addition, the PPIs direct partners are also enriched for COVID-19-related gene sets. HLA alleles prediction from WES data allowed the identification of at least one risk allele in 100% of the MIS-C patients.

**Conclusions:**

This study is the first to explore host MIS-C-associated variants in a Latin American admixed population. Besides expanding the spectrum of MIS-C-associated variants, our findings highlight the relevance of using WES for characterising the genetic interindividual variability associated with COVID-19 complications and ratify the presence of overlapping/convergent mechanisms among MIS-C, KD and COVID-19, crucial for future therapeutic management.

**Supplementary Information:**

The online version contains supplementary material available at 10.1186/s10020-022-00583-5.

## Background

Coronavirus disease 2019 (COVID-19), caused by the severe acute respiratory syndrome coronavirus 2 (SARS-CoV-2), is generally milder in children than in adults (Stokes et al. 2020; Götzinger et al*.* 2020). Nonetheless, in April 2020, a novel entity following SARS-CoV-2 primary infection has emerged in a small subset of children and adolescents and termed “Multisystem Inflammatory Syndrome in Children" (MIS-C) (Riphagen et al. 2020; Verdoni et al. 2020). The Centers for Disease Control definition for MIS-C includes an individual aged < 21 years with a minimum of 24-h of fever > 38.0 °C, severe illness requiring hospitalization with two or more organ systems affected (i.e., cardiac, renal, respiratory, hematologic, gastrointestinal, dermatological, neurological), laboratory evidence of inflammation, laboratory or epidemiological evidence of prior SARS-CoV-2 infection and no alternative diagnosis. The syndrome occurs about three to six weeks after mild or asymptomatic SARS-CoV-2 infection without detectable viral shedding in the respiratory tract (Consiglio et al. 2020; Belot et al. 2020), although SARS-CoV-2 mRNA is noticeable in more than 10% in faecal samples of MIS-C patients up to > 21 days from the presumed contact with the virus (Parodi et al. 2021). The main clinical features of MIS-C include persistent fever, gastrointestinal (GI) symptoms (diarrhea, emesis and abdominal pain), conjunctivitis, cardiac dysfunction, neurological symptoms (headache, confusion, lethargy), shock, rash, lymphopenia, neutrophilia, and hyperferritinemia (Syrimi et al. 2021; Jiang et al. 2022). Some of these features overlap to the symptoms present in the autoimmune Kawasaki disease, and other inflammatory paediatric conditions, such as toxic shock syndrome and hemophagocytic lymphohistiocytosis/macrophage activation syndrome, although the outcomes are distinct (Nakra et al. 2020; Sancho-Shimizu et al. 2021). The geographic distribution of MIS-C is not uniform worldwide, with higher numbers of cases reported in Europe, the Americas, Africa, South Asia, and the Middle East and an expressive scarcity of cases in countries of the East Asia, in which the number of COVID-19 cases was lower (Sancho-Shimizu et al. 2021).

During the past 2 years, some immunopathogenic mechanisms subjacent to MIS-C have begun to be elucidated, including a hyperinflammatory response with increased levels of serum cytokines and endothelial injury, immune dysfunction, a possible role for complement/coagulation, myeloid cell activation and autoimmune dysregulation (Sancho-Shimizu et al. 2021; Ramaswamy et al. 2021; Fraser et al. 2021; Porritt et al. 2021b). Besides, MIS-C patients display uncontrolled neutrophil activation and neutrophil extracellular trap release in the vasculature (Boribong et al. 2021). MIS-C transcriptional signature in blood also partially shares the transcriptional response to SARS-CoV-2 and the Kawasaki disease signature, with enrichment for exhausted CD8* T-cells and CD56^dim^CD57* natural killer cells, suggesting downregulation of natural killer cells and cytotoxic T cell exhaustion response to SARS-CoV-2 infection in MIS-C, with *TBX21*, *TGFBR3*, *C1ORF21*, *S1PR5*, *PRF1*, *MYBL1*, *KLRD1*, *SH2D1B*, and *GZMA* genes as the key drivers of MIS-C pathogenesis (Beckmann et al. 2021). Besides, immunosequencing of MIS-C blood samples revealed expansion of T cell receptor beta variable gene 11-2 (TRBBV11-2), which is correlated to severity and serum cytokine levels, consistent with superantigen triggered immune responses of SARS-CoV-2 and autoimmune signatures (Porritt et al. 2021a; 2021b). Moreover, patients with TRBV11-2 expansion shared human leukocyte antigen class I (HLA-I) alleles A*02, B*35, and C*04, suggesting that MHC class I may mediate TRBV11-2 expansion in a specific HLA-I allele combination (Porritt et al. 2021a). Recently, a multi-omics approach to assess changes of innate and adaptive immune responses between paediatric COVID-19 and MIS-C revealed distinct immunopathological signatures, with MIS-C patients exhibiting elevated levels of soluble biomarkers related to recruitment and activation of monocytes and neutrophils, matrisome activation, vascular endothelium injury, and increased levels of circulating spike protein with no correlation with SARS-CoV-2 PCR status (Sacco et al. 2022). Besides, structure-based computational models demonstrated that the SARS-CoV-2 spike (S) glycoprotein exhibits a high-affinity motif for binding T-cell receptors and may form a ternary complex with major histocompatibility complex class II (MHCII) molecules. Finally, the interaction between SARS-CoV-2 and T cells could be strengthened by the SARS-CoV-2 variant D839Y/N/E (Cheng et al. 2020), which could point to a viral genetics’ contribution to MIS-C development.

It remains elusive, however, why some children previously infected with different SARS-CoV-2 strains develop MIS-C and most do not, suggesting that rare single-gene host genetic variants may have an essential role in increasing the susceptibility to MIS-C. Few studies exploring potential monogenic causes of MIS-C were conducted so far and, although Latino individuals appear to be overrepresented in epidemiological data of MIS-C in the United States studies, in which ancestry was reported (reviewed in Sancho-Shimizu et al. 2021), no whole exome sequencing (WES) study in patients with MIS-C from admixed populations of Latin America has been already conducted.

Herein, we searched for very rare (< 1%) or unique monogenic variants (single nucleotide variants—SNVs or InDels) that could predispose to SARS-CoV-2-triggered hyperinflammation or post-infectious immune/autoimmune dysregulation in a cohort of healthy Brazilian patients with MIS-C. Elucidating the risk factors for MIS-C following SARS-CoV-2 infection is crucial for possible preventive measures and patients’ management and prognosis concerning SARS-CoV-2 and other emergent viruses.

## Methods

### Study participants

A series of 16 children with MIS-C, diagnosed according to the World Health Organization criteria (WHO 2020), was referred to the Paediatric Intensive care unit from Pedro Ernesto University Hospital at State University of Rio de Janeiro and to the Pediatric Rheumatology Service from Instituto de Puericultura e Pediatria Martagão Gesteira at Federal University of Rio de Janeiro, Brazil, from May 2020 to July 2021. All MIS-C patients tested positive for COVID-19 by anti-SARS-CoV-2 antibodies (rapid test or serology) or RT-PCR, with exception of patients EXOC3 and EXOC11, who were non-reagent on serology tests and EXOC17, who was not individually tested. However, the three patients had direct contact with their parents infected by SARS-CoV-2, contemplating the MIS-C WHO criteria (WHO 2020). Two additional patients with MIS-C (EXOC8 and EXOC9) were ruled out from the study, due to insufficient DNA quality. The Institutional Ethics Committees approved the research protocols (CAAE 0135320.0.0000.5259 and 33040420.3.0000.5264) and written informed consent was obtained from the participants and their parents.

### DNA extraction and whole exome sequencing

Peripheral blood samples from MIS-C children were collected after recovery using Tempus™ Blood RNA Tube (Thermo Fisher Inc.) and DNA samples were extracted using Flexigene Kit or QIAamp DNA Blood mini kit (QIAGEN). Sequencing libraries were prepared using the QIAseq Human Exome Kit (Qiagen), according to the manufacturer's protocol. Sequencing was performed using Illumina NextSeq® 500/550 High Output Kit v2 (300 cycles), generating 2 × 149 bp paired-end reads with depth coverage of at least ×100. The short reads were mapped to the human reference genome (GRCh38/hg38) using Bowtie2 version 2.3.4.1 (Langmead and Salzberg 2012). SAM files were converted into BAM files, sorted and filtered by MAPping Quality (MAPQ > 30) using SAMtools version 1.3 (Li et al. 2009; Langmead and Salzberg 2012). MarkDuplicates from Picard tools version 2.18 (http://picard.sourceforge.net/) was applied to mark duplicate reads. SNVs/InDels variants calling was performed using the HaplotypeCaller tool from Genome Analysis Toolkit (GATK) version 4.1 (DePristo et al. 2011).

### Variants prioritization

The prioritization of potentially pathogenic variants related to a high MIS-C risk was performed according to Vianna et al. (2020) with modifications. The filtered variants’ features included (a) sequencing and mapping quality over 30 and read depth over 10; (b) non-synonymous or splice sites variants with moderate or high predicted functional impact according to Variant Effect Predictor (Ensembl) (McLaren et al. 2016); (c) SIFT and/or Polyphen values between 0–0.15 and 0.85–1.0, respectively for coding non-synonymous single nucleotide variants; (d) global minor allele frequency less than 0.01 in at least one populational database (1000 Genomes; ESP6500; Exome Aggregation Consortium—ExAC; The Genome Aggregation Database—GnomAD; and the Brazilian genomic variants database ABraOM).

For better exploring the pathogenic and conservation context of the filtered variants, Varsome (Kopanos et al. 2019) and Variant Effect Predictor (Ensembl) (McLaren et al. 2016) were used for accessing additional computational predictors, including Mutation Taster, Mutation Assessor, M-CAP, Revel, PROVEAN, MetaLR, MetaSVM, MetaRNN, CADD, LRP, GERP++, FATHMM, PhastCons, PhyloP, and Splice Al. OMIM (https://omim.org/), ClinVar (https://www.ncbi.nlm.nih.gov/clinvar/), and Orphanet (https://www.orpha.net/consor/cgi-bin/index.php) were used to search for association with known diseases and inheritance patterns. Only variants with zygosity profiles consistent with the reported inheritance pattern of the related genes were selected. Gene Ontology and KEGG were used to provide functional or pathway annotation for the selected genes. Finally, filtered variants were classified according to the guidelines of the American College of Medical Genetics (ACMG) (Richards et al. 2015). Then, the filtered variants were validated by Sanger sequencing.

### HLA prediction

The HLA alleles were predicted from WES samples with the HLAminer tool (Warren et al. 2012), using the genomic sequences of classes I and II HLA retrieved from the IMGT/HLA database in FASTA format. WES sequences in FASTQ format were mapped against chromosome 6 (GRCh38) using bowtie2 (Langmead and Salzberg 2012) to direct analysis to the chromosome of the HLA *loci*. The sequences recovered were assembled into 200 bp contigs using the TASR tool (Warren and Holt 2011) and aligned to HLA reference sequences using BLAST (Basic Local Alignment Tool). Aligned sequences were then used to predict HLA alleles, including HLA class I (HLA-A, HLA-B, HLA-C, HLA-G, HLA-E, HLA-F) and HLA class II [HLA-DP (DPA1, DPB1, DPB2), HLA-DQ (DQA1, DQA2, DQB1), HLA-DR (DRA, DRB1, 3, 4, 5)].

### Integrative bioinformatic analysis

The context of the filtered variants was analyzed at the functional domain level. The annotation of functional domains was retrieved through the Pfam 32.0 platform (El-Gebali et al. 2019) and the protein architecture was represented with IBS (Liu et al. 2015). The STRING v.11.5 database was used to recover the Protein–Protein Interactions (PPIs) of each mutated gene, and Cytoscape software 3.7.1 (Shannon et al. 2003) was applied to plot the network. Next, the retrieved genes were cross-referenced with those in a dataset of 19 genes, in which variants or abnormal mRNA expression were previously associated with MIS-C (Lee et al. 2020; Chou et al. 2021; Beckmann et al. 2021; Abolhassani et al. 2022; Vagrecha et al. 2022) and 14 genes for which genome-wide studies found an association with Kawasaki disease (Sancho-Shimizu et al. 2021) (Additional file 1: Table S1). Direct interactions with a confidence score > 0.4 were considered for further enrichment analysis by the web server EnrichR (https://maayanlab.cloud/Enrichr/). The gene-set libraries used for enrichment analysis were KEGG 2021 Human, GO Biological Process 2021, GO Molecular Function 2021, GO cellular Component 2021, Jensen Tissues, the scRNA-seq database PanglaoDB Augmented 2021, and COVID-19-related gene sets 2021. Only the top ten terms with an adjusted p-value < 0.05 were retrieved. Additional file 7: Fig. S1 illustrates a workflow of the methodology used in the study.

## Results

The mean age of the MIS-C patients was 7.82 ± 3.94 years (range: 1 year and 3 months–14 years old) with a male predominance (68.7%). Obesity was the most prevalent comorbidity (18.7%) and one patient (EXOC1) had a diagnosis of Kawasaki disease in 2017. None of the patients had been vaccinated for SARS-CoV-2 prior to MIS-C onset. Hospitalisation was not required for two cases (EXOC1 and EXOC5), while five cases were hospitalised out of intensive care units (ICU). For those admitted to ICU, the period of stay ranged between 2 and 19 days. The main onset symptoms included fever (100%), cutaneous rash (81.2%), abdominal pain (75%), conjunctivitis (56.2%), hypotension (43.7%), and shock (43.7%). The most common clinical laboratory findings at the acute MIS-C stage included elevated C-reactive protein (100%) and d-dimer (93.7%), anaemia (87.5%), thrombocytosis (56.2%), hyperferritinemia (50%), elevated fibrinogen (50%), and neutrophilia (50%) (Table 1).

**Table 1:** Summary features seen in the Brazilian MIS-C cohort

Feature (patient code)	1	2	3	4	5	6	7	10	11	12	13	14	15	16	17	18
Age (years)	7	9	3	1	11	1	1	1	9	8	12	10	6	11	9	7
Gender	M	M	M	M	F	F	M	M	F	F	M	F	M	M	M	M
Comorbidities	KD	Obs	WI, LI	−	Obs	−	−	−	Obs	−	−	−	−	−	−	−
Time after COVID-19 (weeks)	3	4	2	4	4	3	3	3	4	9	6	6	7	6	5	2
Length of hospital stay (days)	0	11	6	6	0	6	12	9	11	19	9	8	12	9	9	18
Admission to ICU*	−	+	−	−	−	−	+	−	−	+	+	+	+	+	+	+
Fever	+	+	+	+	+	+	+	+	+	+	+	+	+	+	+	+
Rash	+	−	+	+	+	+	+	+	+	+	+	−	−	+	+	+
Conjunctivitis	+	−	+	+	+	−	+	−	−	+	−	+	−	+	+	−
Cardiovascular dysfunction	−	+	−	−	−	−	+	−	−	+	+	−	+	−	−	+
Abdominal pain	−	+	−	+	+	−	+	−	+	+	+	+	+	+	+	+
Headache	−	−	−	−	−	−	−	−	−	+	+	−	−	−	−	−
Confusion	−	−	−	−	−	−	−	−	−	+	−	−	−	+	−	−
Lethargy	−	−	−	−	−	−	−	−	−	+	−	−	−	+	−	−
Hypotension	−	+	−	−	−	−	−	−	−	+	+	−	+	+	+	+
Allucinations	−	−	−	−	−	−	−	−	−	+	−	−	−	−	−	−
Renal impairment	−	−	−	−	−	−	−	−	−	−	−	−	−	−	−	−
Respiratory dysfunction	−	+	−	−	−	−	+	−	−	+	+	−	−	+	−	−
Shock	−	+	−	−	−	−	+	−	−	+	+	−	+	+	+	−
Elevated CRP	+	+	+	+	+	+	+	+	+	+	+	+	+	+	+	+
Hyperferritinemia	NA	+	−	NA	NA	+	−	−	−	+	+	−	+	+	+	+
Elevated d-dimer	+	+	+	+	+	+	−	+	+	+	+	+	+	+	+	+
Lymphopenia	−	+	−	−	−	−	−	−	−	+	−	−	−	−	−	NA
Neutrophilia	−	+	−	+	−	−	−	−	−	+	+	−	+	+	+	+
Anaemia	+	+	−	+	−	+	+	+	+	+	+	+	+	+	+	+
Thrombocytosis	+	−	−	+	−	−	−	−	−	+	+	+	+	+	+	+
Increased APTT	−	+	+	+	+	−	+	−	−	+	−	−	−	−	−	+
High fibrinogen	NA	+	+	+	NA	+	−	+	+	+	−	−	−	−	−	+
Coronary dilatation	−	−	−	−	−	−	−	−	−	−	−	+	+	+	+	+

(+): presence; (−): absence; M: male; F: female; NA: not available; CRP: C-Reactive Protein; KD: previous Kawasaki disease (2017); Obs: obesity; WI: Wheezing infant; LI: lactose intolerance; ICU: intensive care unit. APTT: activated partial thromboplastin time. *Time of ICU stay for patients 2 and 7 was equal to 4 and 2 days, respectively

All patients recovered from MIS-C after different treatment strategies, such as intravenous immunoglobulin, AAS, immune-modulatory agents and systemic steroids. Supportive care (vasoactive medication, invasive mechanical ventilation) was also applied when necessary. Patients EXOC14, EXOC15, EXOC16 and EXOC18 developed hypothyroidism, attention deficit hyperactivity disorder (ADHD), migraine and Reentrant Supraventricular Tachycardias (RST), respectively during the follow-up.

WES revealed 10 potentially causative variants (5 missense, 2 nonsense, 1 splice acceptor, 1 frameshift deletion and 1 frameshift duplication) that could be associated with a higher susceptibility risk of MIS-C in 9 patients (Table 2; Additional file 2: Tables S2 and Additional file 3: Table S3, Additional file 7: Fig. S2). All these potentially causative variants are very rare (Minor Allele Frequency < 0.01), were predicted to be damaging by the different algorithms used and were classified as pathogenic, likely pathogenic or variants of unknown significance (VUS), according to the ACMG parameters (Table 2). These variants have been submitted to the National Center for Biotechnology Information (NCBI, BioProject ID: PRJNA848757). Other filtered variants with no clear relation with MIS-C and/or incompatible inheritance patterns are reported in Additional file 4: Table S4. No very rare relevant variant was found in patient EXOC17.

**Table 2:** Potentially causative variants identified by WES in children with MIS-C

Patient	Gene (Location)	Mutation type	Mutation description (GRCh38/hg38)	Status	MAF (GnomAD genomes/ ABraOM)	Gene function	Inheritance pattern (OMIM) *	Conclusion (ACMG parameters) **
EXOC1	*FREM1* (9p22.3)	Nonsense	Chr9:14808083G > T NM_001379081.2 c.2945C > A:p.Ser982* r377212852	Heterozygous	0.003%/NA	FRAS1 related extracellular matrix 1	AR/AD	Pathogenic (PVS1, PM2)
EXOC3	*MPO* (17q22)	Splice acceptor	Chr17:58270865 T > G NM_000250.2 c.2031-2A > C rs35897051	Heterozygous	0.5%/0.2%	Myeloperoxidase	AR/AD	Pathogenic (PVS1, PM2, PP5, PS3)
	*POLG* (15q26.1)	Missense	Chr15:89323423A > G NM_002693.3 c.2246 T > C:p.Phe749Ser rs202037973	Heterozygous	0.02%/NA	DNA polymerase gamma, catalytic subunit	AR/AD	VUS (PM2, PP3)
EXOC5	*C6* (5p13.1)	Frameshift	Chr5:41176504G > T NM_000065.5 c.1138delC:p.Gln380fs rs375762365	Heterozygous	0.2%/0.04%	Complement C6	AR with reported cases of heterozygous case with reduced C6 levels	Pathogenic (PVS1, PM2, PP5)
EXOC6	*ABCA4* (1p22.1)	Missense	Chr1:94021934A > G NM_000350.3 c.4685 T > C:p.Ile1562Thr rs1762111	Heterozygous	0.01%/0.04%	ATP binding cassette subfamily A member 4	AR/AD	VUS (PM2, PP2, PP3)
EXOC13	*ABCA4* (1p22.1)	Missense	Chr1:94001992C > G NM_000350.3 c.6149G > C:p.Val2050Leu rs41292677	Heterozygous	0.4%/0.3%	ATP binding cassette subfamily A member 4	AR/AD	Pathogenic (PM1, PM2, PP3, PP2, PP5)
EXOC14	*ABCA4* (1p22.1)	Missense	Chr1:94098885C > A NM_000350.3 c.677G > T:p.Arg226Leu rs144310835	Heterozygous	0.2%/0.08%	ATP binding cassette subfamily A member 4	AR/AD	VUS (PM2, PP2)
EXOC15	*C9* (5p13.1)	Nonsense	Chr5:39342112G > T NM_001737.5 c.162C > A:p.Cys54* rs34000044	Heterozygous	0.06%/0.04%	Complement C9	UN	Pathogenic (PVS1, PM2, PP5)
EXOC16	*ABCC6* (16p13.11)	Missense	Chr16:16154974G > A NM_001171.6 c.3940C > T:p.Arg1314Trp rs63750759	Heterozygous	0.08%/0.1%	ATP binding cassette subfamily C member 6	AR/AD	Pathogenic (PP5, PM1, PM2, PM5, PP2, PP3)
EXOC18	*BSCL2* (11q12.3)	Frameshift	Chr11:62694680G > GT NM_001122955.4 c.517dupA:p.Thr173fs rs786205071	Heterozygous	NA/NA	BSCL2 lipid droplet biogenesis associated, seipin	AR/AD	Pathogenic (PVS1, PM2, PP5)

VUS: variant of unknown significance; NA: not available; *According to OMIM—Online Mendelian Inheritance in Man®; **According to ACMG guidelines (Richards et al, 2015) and Varsome

The variants p.Ser982* (*FREM1*), p.Gln380fs (*C6*), p.Val2050Leu (*ABCA4*), p.Cys54* (*C9*), p.Arg1314Trp (*ABCC6*), and p.Thr173fs (*BSCL2*) are contained in regions annotated as functional domains in the canonical proteins (Additional file 7: Fig. S3). Such variants occur in the Cadherin_3 domain (PF16184; *FREM1* gene), MACPF domain (PF01823; *C6* gene), TSP_1 domain (PF00090; *C9* gene), in the ABC_tran domain (PF00005; *ABCA4* and *ABCC6* genes), and Seipin domain (PF06775; *BSCL2* gene). The changes observed in *FREM1* and *C9* resulted in premature stop codons, leading to proteins with 45% and 9% of the original size, respectively. In the FREM1 protein, the premature stop occurs before six regions of Cadherin_3, one Calx-beta domain (PF03160) and Lectin_C domain (PF00059). Premature stop at *C9* occurs in the initial portions of the domain harbouring the variant (TSP_1), resulting in its loss, in addition to Ldl_recept_a (PF00057) and MACPF (PF01823). Three other variants are outside functional domains: p.Phe749Ser (*POLG*), p.Arg226Leu and p.Ile1562Thr (*ABCA4*). Considering all the missense changes, only p.Val2050Leu (*ABCA4*) concerns a change by an amino acid with similar properties, while the others (*POLG* p.Phe749Ser; *ABCA4* p.Arg226Leu and p.Ile1562Thr; *ABCC6* p.Arg1314Trp) modify some properties. The p.Phe749Ser variant (*POLG*) corresponds to a non-polar aromatic amino acid change by another hydroxylic and polar one. The two changes involving Arginine (*ABCA4* p.Arg226Leu and *ABCC6* p.Arg1314Trp) result in a change of a polar and positive amino acid by a non-polar one. Besides, the p.Ile1562Thr (*ABCA4*) variant replaces a non-polar aliphatic amino acid with a polar, hydroxylic one. Due to its longer chain, p.Val2050Leu (*ABCA4*) can alter the hydrophobicity, even involving two similar amino acids.

Regarding the unique and shared interactions retrieved by STRING, the analysis highlighted 891 proteins in the network (FREM1: 53, MPO: 274, POLG: 156, C6: 91, C9: 106, ABCA4: 131, ABCC6: 39, and BSCL2: 124). MPO showed association with the products of three Kawasaki disease genes (*FCGR2A*, *CASP3*, and *LTA*) and one related to MIS-C (*CYBB*). The *C9* gene product showed interactions with the product of two MIS-C-related genes (*PRF1* and *GZMA*) and interaction with C6. In addition, we observed interactions between ABCA4 and ABCC6 (Additional file 7: Fig. S4).

The enrichment analysis through KEGG revealed complement/coagulation cascades, hematopoietic cell lineage and coronavirus disease among the most ten prevalent terms. GO enrichment demonstrated neutrophil-mediated immunity, neutrophil degranulation, neutrophil activation, regulation of complement activation, regulation of immune effector process, regulation of humoral immune response, cytokine-mediated signalling pathway, inflammatory response and cellular response to cytokine stimulus as the main enriched biological processes. GO enrichment for Molecular Function evidenced DNA polymerase activity, serine type endopeptidase/peptidase activity, and cytokine activity, while GO enrichment for Cellular Component found secretory granule lumen and intracellular organelle lumen as the most enriched terms. Jensen tissues enrichment pointed to the immune system as the most enriched tissue, whereas PanglaoDB Augmented 2021 revealed neutrophils and monocytes as the most enriched cell types. Finally, analysis based on COVID-19-related gene sets highlighted different enriched terms for human (PBMC, lung, organoids) or murine (liver, lung) cells (Additional file 7: Fig. S5).

In addition to rare variant analyses, classic HLA alleles (HLA-A, HLA-B, HLA-C, HLA-DP, HLA-DQ and HLA-DR) and non-classic HLA-F, HLA-G, and HLA-H alleles were predicted from WES data at four-digit resolution. All patients were carriers of at least one potential risk allele (grey highlights) (Additional file 5: Table S5).

## Discussion

The host genetic background of an individual underwrites the susceptibility and response to viral infection and its outcomes. However, host monogenic predisposing factors to MIS-C remain elusive. In this study, we evaluated 16 Brazilian individuals who had MIS-C for monogenic very rare variants that could predispose them to this clinical condition. The median age of children affected by MIS-C, clinical features and prevalence of comorbidities are in accordance with those of previously published cohorts (Ahmed et al. 2020; Feldstein et al. 2020, Lee et al. 2020; Chou et al. 2021; Sancho-Shimizu et al. 2021). However, similar to Vagrecha et al. (2022), we found a clear male preponderance (68.7%) in our MIS-C cohort. The male sex bias is also evident for COVID-19 hospitalization and death, compared to females (The Sex, Gender and COVID-19 Project, 2022). Besides the role of the X-linked *ACE2* gene, coding for the SARS-CoV-2 receptor, Takahashi et al. (2020) reported that female patients have more robust T cell activation during SARS-CoV-2 infection, and male patients have higher plasma levels of innate immune cytokines (Takahashi et al. 2020). Although the SARS-CoV-2 strains were not discriminated in MIS-C patients during COVID-19, we suppose that almost all patients were infected by the B.1.1.33 lineage, the most prevalent established strain circulating during the first 8 months (March to October 2020) of pandemics in our state, according to genomic surveillance data (Francisco Junior et al. 2021). Patient EXOC18, however, is most likely to have been infected by the P.1 strain, since his hospital admission occurred in March 2021, when this lineage was assumed as the predominant (Francisco Junior et al. 2021).

Few studies focusing on monogenic causes for MIS-C development have been conducted (Additional file 6: Table S6). Lee et al. (2020) reported one adolescent with immune thrombocytopenia and autoimmune haemolytic anaemia who recovered from SARS-CoV-2 infection and developed MIS-C. WES in this patient revealed a heterozygous loss-of-function variant in the suppressor of cytokine signalling 1 (*SOCS1*) gene, an essential regulator of type I and type II IFN signalling (Lee et al. 2020). Subsequently, the group expanded the analysis to more 17 patients with MIS-C, from which two boys were described as having hemizygous deleterious variants in the X-linked inhibitor of apoptosis (*XIAP*) and cytochrome b-245 beta chain (*CYBB*) genes. *XIAP* plays roles in cell survival, activation, and negative regulation of the NLRP3 inflammasome (Jost et al. 2020), in a manner that patients with *XIAP* loss-of-function variants are at risk for virally triggered hemophagocytic lymphohistiocytosis (Marsh et al. 2010). *CYBB* encodes the p91^phox^ subunit of the NADPH oxidase, essential for the phagocytic oxidative burst. Loss of NADPH oxidase impairs the generation of reactive oxygen species, which inhibit type I interferon signalling, promoting proinflammatory macrophage phenotype (Chou et al. 2021). Furthermore, rare inborn errors of immunity (IEIs) perturbing the immune response to SARS-CoV-2 and dysregulation of type I interferons (IFN)s immunity were proposed to underlie the MIS-C pathogenesis in some children (Sancho-Shimizu et al. 2021; Abolhassani et al. 2022). In this sense, a 3-year-old boy with critical COVID-19 pneumonia and MIS-C harbouring a large, homozygous frameshift deletion in the *IFNAR1* gene was reported, confirming the role of impaired type I IFN immunity in MIS-C in concomitance to critical COVID-19 pneumonia (Abolhassani et al. 2022). Finally, Vagrecha et al. (2022) used a monogenic autoimmunity panel to investigate genetic variations in 109 genes crucial to immune regulation amongst children with MIS-C, from whom 25.4% exhibited rare heterozygous variants of unknown significance in primary hemophagocytic lymphohistiocytosis genes (*LYST*, *STXBP2*, *PRF1*, *UNC13D*, *AP3B1*) or the hemophagocytic lymphohistiocytosis-associated *DOCK8* gene (Vagrecha et al. 2022).

We consider that very rare monogenic variants, which were clinically silent previous to COVID-19, could propitiate a less effective immune response to infection or trigger the inflammatory response or a delayed hyperimmune response to SARS-CoV-2, leading to a higher susceptibility to manifest MIS-C. Indeed, both MIS-C and severe COVID-19 have been classified as hyperinflammatory cytokine storm syndromes (Mehta et al. 2021; Noval Rivas et al. 2021).

Patient EXOC1 has a heterozygous nonsense variant in the Fras-relate extracellular matrix 1 (*FREM1*) gene (NM_001379081.2; c.2945C > A:p.Ser982*), classified as pathogenic, according to ACMG guidelines with no submissions to ClinVar. Host defense against infection is induced by Toll-like and interleukin (IL)-1 receptors and is controlled by the transcription factor NF-kappaB (Zhang et al. 2010). The *FREM1* gene encodes a full-length isoform that acts as an extracellular matrix protein in epidermal differentiation. Besides, it produces a shorter splice variant isoform named Toll-like/interleukin-1 receptor regulator (TILRR) that is a co-repressor of the interleukin 1 receptor family and acts as a major modulator of many genes in the NF-kappaB signal transduction pathway, promoting the production of multiple pro-inflammatory cytokines/chemokines and modulating inflammatory process (Kashem et al. 2021a, b). So, we believe that the *FREM1* variant may predispose to MIS-C due to a less effective immune response.

Patient EXOC3 has a heterozygous pathogenic variant in the myeloperoxidase (*MPO*) gene (NM_000250.2; c.2031-2A > C) affecting a splice acceptor site. MPO is a heme-containing peroxidase predominantly expressed in neutrophils that is part of the innate immune system. Neutrophils play a crucial role in host defense against pathogens through the generation of reactive oxygen species. Upon activation of neutrophils in peripheral blood and tissues, MPO is released into the phagolysosomal compartment and catalyses the formation of reactive oxygen intermediates, including hypochlorous acid. MPO release happens concomitantly with the assembly of an NADPH oxidase (NOX) complex on the inner phagolysosomal membrane, producing superoxide radicals (O2•-) from molecular O2, called the oxidative burst. Dismutation of O2•- generates the H2O2 required for MPO activity and pathogen killing (Hawkins and Davies 2021). Individuals with MPO deficiency are less efficient to kill intracellular pathogens and also experience higher rates of infections and chronic inflammatory conditions (Odobasic et al. 2016; Strzepa et al. 2017). However, increased susceptibility to severe infection in patients with MPO deficiency is documented so far in bacterial infection. It should be possible that the relation between MPO deficiency and susceptibility to develop MIS-C is related to the effect of oxidative stress as a potent controller of the inflammatory response through the induction of oxidant-mediated elimination of activated T cells and macrophages. Furthermore, a significant positive correlation between increased neutrophil and cardiac dysfunction, inflammation, and disease severity was reported in MIS-C (Syrimi et al. 2021).

Besides the *MPO* variant, patient EXOC3 has also a heterozygous missense variant in the DNA polymerase gamma catalytic subunit (*POLG*) gene (NM_002693.3 c.2246T > C:p.Phe749Ser), classified as a VUS, according to ACMG guidelines. Mitochondrial dysfunction is considered an important driver of the mammalian innate immune response, having a dual opposite role in facilitating antibacterial immunity by generating reactive oxygen species (ROS), and contrariwise promoting inflammation following cellular damage and stress. Mitochondrial DNA is an agonist of Toll-like receptor 9 (TLR9), NOD-like receptor family pyrin domain containing 3 (NLRP3), and cyclic GMP-AMP synthase (cGAS). The cGAS-stimulator of interferon genes (STING) axis is recognized as a major driver of type I interferon (IFN-I) and inflammatory responses to nuclear and mitochondrial genome instability, and *Polg* mutator homozygous mice exhibit a hyperinflammatory innate immune status that is driven by chronic engagement of the cGAS-STING–IFN-I axis (Lei et al. 2021). Heterozygous *Polg* (D257A) knock-in mice demonstrated tissue-specific, age-dependent accumulation of multiple mtDNA deletions in the brain and muscles which was likely to result in neuromuscular symptoms (Fuke et al. 2014). In humans, *POLG* pathogenic variants in both recessive (homozygous mutation or compound heterozygous mutation) and dominant models can lead to different clinical spectrums (OMIM, 2022; Tang et al. 2011). The rs202037973 T > C variant has conflicting interpretations of pathogenicity in ClinVar [pathogenic (2); likely pathogenic (5); uncertain significance (2)]. So, we cannot discard a synergistic involvement of both *MPO* and *POLG* variants in patient EXOC3 concerning the MIS-C development risk.

EXOC5 and EXOC15 patients have a frameshift (NM_000065.5; c.1138delC:p.Gln380fs) and a nonsense (NM_001737.5; c.162C > A:p.Cys54*) heterozygous variants in the complement *C6* and *C9* genes, respectively. For both genes, the variants found were classified as pathogenic according to ACMG guidelines. The complement system is considered a vital part of the innate immune system and modulates adaptive immunity. The recognition of pathogens or associated molecular patterns by complement proteins triggers a cascade of events that culminate in the engagement of complement receptors on the surface of immune cells. The signalling pathways shotted by the receptors regulate the type and extent of immune responses. Moreover, complement receptor-mediated signalling is related to homeostatic and pathologic T-cell responses, having a role in adaptive immunity (Reis et al. 2019). Systemic complement activation of C9 and C5b-9 was reported to be a common feature in MIS-C patients, which could explain the endothelial damage and shock seen in such syndrome. After treatment, a decrease in their levels is noted (Syrimi et al. 2021). Complement dysregulation has been also observed in severe COVID-19 (Holter et al. 2020). Generally, complement deficiencies are inherited as autosomal recessive traits (homozygous or compound heterozygous), except for properdin (X-linked recessive pattern), *C1* inhibitor (autosomal dominant pattern) and *C9*, whose inheritance pattern is undetermined, according to OMIM. However, for the *C6* gene, heterozygous carriers have already been described in the literature as having C6 deficiency (Würzner et al. 1995). Recently, a heterozygous nonsense variant (c.1062C > G/p.Y354*) was reported in a paediatric patient with meningococcal disease, leading to low levels of serum C6, far below the mean normal level (Zhang et al. 2021). To date, 15 pathogenic variants in the *C6* gene have been identified in patients with C6 deficiency, including the frameshift c.1138delC in exon 8/18 (reviewed in Zhang et al. 2021). In the same way, individuals heterozygous for a *C9* nonsense pathogenic variant have already been described (Horiuchi et al. 1998). So, given the importance of complement genes to innate and adaptive immunity, we considered that *C6*/*C9* variants have a role in the MIS-C development due to a deficient immune response.

Curiously, 4/16 (25%) patients have very rare variants in ATP-binding cassette (ABC) superfamily genes (Dean et al. 2022). Patient EXOC 16 exhibited a heterozygous pathogenic missense variant in the ATP-binding cassette subfamily A member 6 (*ABCC6*) gene and three other patients (EXOC6, EXOC13, and EXOC14) in our cohort exhibit heterozygous missense variants (one pathogenic and two VUS) in the ATP-binding cassette subfamily A member 4 (*ABCA4*) gene. The ABC superfamily consists of membrane proteins that transport a wide variety of substrates across membranes and are divided into seven distinct subfamilies (ABC1, MDR/TAP, MRP, ALD, OABP, GCN20, White). For the *ABCA6* gene, a member of the MRP subfamily, the major expression tissues at mRNA level are liver and gallbladder, kidney and urinary bladder, pancreas, and gastrointestinal tract, with enrichment for classical monocytes and neutrophils in immune cells (The Human Protein Atlas 2022). At the mRNA level, *ABCA4*, a member of the ABC1 subfamily, is predominantly expressed in the brain and eyes, whereas at the protein level, the major expression occurs in the eyes, kidneys and urinary bladder (The Human Protein Atlas 2022). *ABCA4* gene is amongst the 500 genes down-regulated by SARS-CoV-2 in human Organoids from GSE154613 (COVID-19-related gene sets 2021, EnrichR). Besides, the global network for human proteins interacting with *ACE2* recovered from the Human Base COVID-19 database (https://hb.flatironinstitute.org/covid19) clustered into 10 functional modules, from which ABCC6 protein is present in the co-expression module 6, enriched in urate metabolic process, organic anion transport, and small molecule biosynthetic process.

Patient EXOC18 has a heterozygous frameshift variant (c.517dupA:p.Thr173fs) in the BSCL2 lipid droplet biogenesis associated, seipin (*BSCL2*) gene. Lipid droplets are specialized organelles, in which eukaryotic cells store large amounts of esterified neutral lipids (triacylglycerols/TAGs and sterol esters/SEs). Phagocytosis of invading pathogens by specialized cells, such as macrophages and neutrophils, and progression/maturation of pathogen-containing phagosomes, a key component of the innate immune response, occurs in parallel with the accentuated formation of lipid-rich organelles. Seipin plays crucial roles in lipid droplets biogenesis and dynamics in various cell types, including immune cells, in which lipid droplets synthetize and store inflammatory mediators, considered structural markers of inflammation. The interaction of lipid droplets with phagosomes containing pathogens occurs in response to infections, influencing the outcome or survival of the pathogen within host cells (Melo and Dvorak 2012). Thus, seipin could interfere in the capacity of macrophages to respond to pathogens through the lipid droplet metabolism and one-third of patients with congenital generalised lipodystrophy, a recessive condition caused by *BSCL2* variants, died of infectious disease (Zhou et al. 2022). Although patient EXOC18 had no noticed lipodystrophy previous to MIS-C, *BSCL2* variants are also described in dominant clinical conditions (OMIM), which could explain the higher susceptibility to MIS-C. Furthermore, global lipodystrophic *Bscl2*−/−mice exhibit hypertrophic cardiomyopathy with reduced cardiac steatosis, and mice with cardiac-specific deletion of *Bscl2* developed systolic dysfunction with dilation, through excessive lipid catabolism (Zhou et al. 2022). Curiously, patient EXOC18 presented Reentrant Supraventricular Tachycardias (RST) phenotype during the follow- up.

To better elucidate the functional context, in which the variants in *FREM1*, *MPO*, *POLG*, *C6*, *C9*, *ABCA4*, *ABCC6*, and *BSCL2* genes, we used a global network of PPIs. Further analysis of the direct partners evidenced proteins that were previously linked to MIS-C or Kawasaki disease. Besides, enrichment analysis highlighted significant immune and inflammatory response terms in multiple datasets concerning pathways, biological processes, molecular function, cellular components, tissues, and cell types. Furthermore, enrichment for COVID-19-related gene sets revealed proteins enriched in different experiments from both human and murine cells. Taken together, these findings reinforce the role of the mutated genes in MIS-C and ratify the existence of overlapping or convergent immune and inflammatory mechanisms underlying MIS-C, Kawasaki disease, and COVID-19.

Besides the MIS-C-related variants described, we found a heterozygous likely pathogenic variant in aldehydrogenase 7 family member A1 (*ALDH7A1*; c.34delG:p.Ala12fs) in patient EXOC10 that is noteworthy. The aldehyde dehydrogenases (ALDHs) are a superfamily of NADP^+^‐dependent enzymes that metabolize endogenous and exogenous aldehydes to corresponding carboxylic acids. Recent evidence suggests that ALDH has a role in regulatory T (Treg) cells induction and function. Treg cells are components of the immune system that promote immune tolerance and prevent aberrant immune responses to beneficial or non-harmful pathogens (Bazewicz et al. 2019). Particularly, ALDH7A1 was reported to attenuate reactive aldehyde and oxidative stress-induced cytotoxicity (Brocker et al. 2011). So, as oxidative stress is present in MIS-C patients, we cannot completely discard the involvement of a subclinical less effective ALDH7A1 enzyme over MIS-C risk, even that *ALDH7A1* variants were only related to a clinical trait (OMIM 107323) in a homozygous or compound heterozygous status.

The variants identified in previous MIS-C studies were not detected in our cohort and there are no overlapping variants among previous studies. The absence of common variants in different studies could be mainly due to the rarity of the variants, the small number of studies focusing on host genetic susceptibility to MIS-C and discrepancies concerning patients’ selection and experimental design. However, in our study, there is clear a recurrence of variants in genes within the same family, such as *ABCA4*/*ABCC6* and *C6*/*C9*.

We also examined the MIS-C samples for class I and II HLA alleles. Regarding class I alleles, three major genotypes A*02, B*35, and C*04, previously described to increase the risk of MIS-C (Kouo et al. 2021; Mazer et al. 2022; Sacco et al. 2022), were found in ten (62.5%) of our patients (Additional file 5: Table S5). Concerning the HLA class II, risk alleles DRB1*15:01 and DQB1*06:02, observed in children with SARS-CoV-2 infection (Valentini et al. 2021), were predicted in five (31.2%) and eight (50%) of our patients, respectively (Additional file 5: Table S5). So, altogether HLA alleles prediction from WES data of the MIS-C patients allowed the identification of at least one risk allele in 100% of the patients.

The frequencies of some clinical or laboratory findings in our cohort are quite different from what was reported in large non-genetic studies (Feldstein et al. 2020), including a higher proportion of coronary dilatations and thrombocytosis, as well as a lower prevalence of lymphocytopenia. We consider that these discrepancies could be a result of the small sample size or, alternatively, a feature of the Brazilian population. Indeed, the higher proportion of coronary dilatations is in accordance with a multicenter, prospective cohort study, conducted in 17 pediatric intensive care units in five states in Brazil, which revealed that 27% of 56 patients with MIS-C had signs of coronary dilatation (Lima-Setta et al. 2021). Besides, the patients with coronary alterations (all of them hospitalised in ICU) had thrombocytosis, which could be associated with the inflammatory involvement of the vascular endothelium. So, the inclusion of ICU children with a great severity may have caused an overestimation of thrombocytosis.

## Conclusions

Host genetic predisposition knowledge on MIS-C risk remains limited and no study had been conducted in admixed populations of Latin America. We used WES to investigate very rare SNVs/InDels in MIS-C patients and identified ten very rare/unique variants in eight genes as the most promising candidates. Our findings highlight the relevance of using WES for identifying host genetic interindividual variability associated with COVID-19 complications and emphasize the need for COVID-19 prevention, even in children. Besides, characterization of host genetic factors predisposing to MIS-C may reveal biological mechanisms crucial for therapeutic management aiming to modify the worst outcomes.

## Supplementary Information

**Additional file 1: Table S1.** Genes in which sequence variants or abnormal mRNA expression were previously associated with MIS-C (Lee et al. 2020; Chou et al. 2021; Beckmann et al. 2021; Abolhassani et al. 2022; Vagrecha et al. 2022) and genes for which genome-wide studies found association with Kawasaki disease (Sancho-Shimizu et al. 2021).

**Additional file 2: Table S2.** Overview of WES data quality.

**Additional file 3: Table S3.** Sequencing metrics to support the call quality of each potentially causative variant.

**Additional file 4: Table S4.** Additional very rare variants (MAF < 0.01) found in MIS-C patients. No relevant variants were found in patient EXOC17.

**Additional file 5: Table S5.** Prediction of class I and II HLA alleles from WES data.

**Additional file 6: Table S6.** Previous host genetic variants described in patients with MIS-C by whole exome sequencing or immune-gene-panel.

**Additional file 7: Figure S1.** Workflow of the methodology used. **Figure S2.** Electropherograms obtained by Sanger sequencing, confirming the WES results. **Figure S3.** Location of the potentially causative variants in the canonical protein forms obtained from the UniProt Platform. The proteins, proportional to size, the positions of the mutations (lollipops), and the coordinates of the domains annotated in Pfam are represented. Variants identified in *FREM1, C6, C9, ABCC6,* and *BSCL2* are contained in the functional domains of their proteins, whereas for the *ABCA4* variant, two variants are outside functional domains and one is within it. For *POLG*, the variant is located outside functional domains. *MPO* variant was not illustrated, since it is intronic. **Figure S4.** Highly reliable protein–protein interactions network retrieved from the products of the eight genes studied. The red hexagons represent the genes carrying the variants. The circles represent the interactions obtained through the STRING plugin, with the green circles representing genes already related to the MIS-C phenotype and blue circles representing genes already related to Kawasaki disease. **Figure S5.** Enrichment analysis through EnrichR tool of the proteins verified in the PPIs network (FDR < 0.05) against the following gene-set libraries: KEGG 2021 (a); Gene Ontology 2021—biological processes (b), molecular function (c), and cellular component (d); Jensen tissues (e); the scRNA-seq database PanglaoDB Augmented 2021 (f); COVID-19-related gene sets 2021 (g). Top ten elements were sorted by adjusted p-value ranking.

## Data Availability

The variants herein described have been submitted to the National Center for Biotechnology Information (NCBI, BioProject ID: PRJNA848757).

## References

[CR1] Abolhassani H, Landegren N, Bastard P, Materna M, Modaresi M, Du L (2022). Inherited IFNAR1 deficiency in a child with both critical COVID-19 pneumonia and multisystem inflammatory syndrome. J Clin Immunol.

[CR2] Ahmed M, Advani S, Moreira A, Zoretic S, Martinez J, Chorath K (2020). Multisystem inflammatory syndrome in children: a systematic review. EClinicalMedicine.

[CR3] Bazewicz CG, Dinavahi SS, Schell TD, Robertson GP (2019). Aldehyde dehydrogenase in regulatory T-cell development, immunity and cancer. Immunology.

[CR4] Beckmann ND, Comella PH, Cheng E, Lepow L, Beckmann AG, Tyler SR (2021). Downregulation of exhausted cytotoxic T cells in gene expression networks of multisystem inflammatory syndrome in children. Nat Commun.

[CR5] Belot A, Antona D, Renolleau S, Javouhey E, Hentgen V, Angoulvant F (2020). SARS-CoV-2-related paediatric inflammatory multisystem syndrome, an epidemiological study, France, 1 March to 17 May 2020. Euro Surveill.

[CR6] Boribong BP, LaSalle TJ, Bartsch YC, Ellett F, Loiselle ME, Davis JP, et al. Neutrophil Profiles of Pediatric COVID-19 and Multisystem Inflammatory Syndrome in Children. bioRxiv [Preprint]. 2021:2021.12.18.473308. 10.1016/j.xcrm.2022.100848 PMC9676175 36476388

[CR7] Brocker C, Cantore M, Failli P, Vasiliou V (2011). Aldehyde dehydrogenase 7A1 (ALDH7A1) attenuates reactive aldehyde and oxidative stress induced cytotoxicity. Chem Biol Interact.

[CR8] Cheng MH, Zhang S, Porritt RA, Noval Rivas M, Paschold L, Willscher E (2020). Superantigenic character of an insert unique to SARS-CoV-2 spike supported by skewed TCR repertoire in patients with hyperinflammation. Proc Natl Acad Sci U S A.

[CR9] Chou J, Platt CD, Habiballah S, Nguyen AA, Elkins M, Weeks S (2021). Mechanisms underlying genetic susceptibility to multisystem inflammatory syndrome in children (MIS-C). J Allergy Clin Immunol.

[CR10] Consiglio CR, Cotugno N, Sardh F, Pou C, Amodio D, Rodriguez L (2020). The immunology of multisystem inflammatory syndrome in children with COVID-19. Cell.

[CR11] COVID-19 Host Genetics Initiative (2021). Mapping the human genetic architecture of COVID-19. Nature.

[CR12] Dean M, Moitra K, Allikmets R (2022). The human ATP-Binding Cassette (ABC) transporter superfamily. Hum Mutat.

[CR13] DePristo MA, Banks E, Poplin R, Garimella KV, Maguire JR, Hartl C (2011). A framework for variation discovery and genotyping using next-generation DNA sequencing data. Nat Genet.

[CR14] El-Gebali S, Mistry J, Bateman A, Eddy SR, Luciani A, Potter SC (2019). The Pfam protein families database in 2019. Nucleic Acids Res.

[CR15] Feldstein LR, Rose EB, Horwitz SM, Collins JP, Newhams MM, Son MBF (2020). multisystem inflammatory syndrome in U.S. children and adolescents. N Engl J Med.

[CR16] Francisco Junior RDS, Lamarca AP, de Almeida LGP, Cavalcante L, Machado DT, Martins Y (2021). Turnover of SARS-CoV-2 lineages shaped the pandemic and enabled the emergence of new variants in the State of Rio de Janeiro, Brazil. Viruses.

[CR17] Fraser DD, Patterson EK, Daley M, Cepinskas G (2021). Case report: inflammation and endothelial injury profiling of COVID-19 pediatric multisystem inflammatory syndrome (MIS-C). Front Pediatr.

[CR18] Fuke S, Kametani M, Yamada K, Kasahara T, Kubota-Sakashita M, Kujoth GC (2014). Heterozygous Polg mutation causes motor dysfunction due to mtDNA deletions. Ann Clin Transl Neurol.

[CR19] Götzinger F, Santiago-García B, Noguera-Julián A, Lanaspa M, Lancella L, Calò Carducci FI (2020). COVID-19 in children and adolescents in Europe: a multinational, multicentre cohort study. Lancet Child Adolesc Health..

[CR20] Holter JC, Pischke SE, de Boer E, Lind A, Jenum S, Holten AR (2020). Systemic complement activation is associated with respiratory failure in COVID-19 hospitalized patients. Proc Natl Acad Sci U S A.

[CR21] Horiuchi T, Nishizaka H, Kojima T, Sawabe T, Niho Y, Schneider PM (1998). A non-sense mutation at Arg95 is predominant in complement 9 deficiency in Japanese. J Immunol.

[CR22] Jiang L, Tang K, Irfan O, Li X, Zhang E, Bhutta Z (2022). Epidemiology, clinical features, and outcomes of multisystem inflammatory syndrome in children (MIS-C) and adolescents-a live systematic review and meta-analysis. Curr Pediatr Rep.

[CR23] Jost PJ, Vucic D (2020). Regulation of Cell Death and Immunity by XIAP. Cold Spring Harb Perspect Biol.

[CR24] Kashem MA, Li H, Liu LR, Liang B, Omange RW, Plummer FA, Luo M (2021). The potential role of FREM1 and its isoform TILRR in HIV-1 acquisition through mediating inflammation. Int J Mol Sci.

[CR25] Kashem MA, Yuan XY, Li L, Kimani J, Plummer F, Luo M (2021). TILRR (toll-like interleukin-1 receptor regulator), an important modulator of inflammatory responsive genes, is circulating in the blood. J Inflamm Res.

[CR26] Kopanos C, Tsiolkas V, Kouris A, Chapple CE, Albarca Aguilera M, Meyer R, Massouras A (2019). VarSome: the human genomic variant search engine. Bioinformatics.

[CR27] Kouo T, Chaisawangwong W (2021). SARS-CoV-2 as a superantigen in multisystem inflammatory syndrome in children. J Clin Invest.

[CR28] Langmead B, Salzberg SL (2012). Fast gapped-read alignment with Bowtie 2. Nat Methods.

[CR29] Lee PY, Day-Lewis M, Henderson LA, Friedman KG, Lo J, Roberts JE (2020). Distinct clinical and immunological features of SARS-CoV-2-induced multisystem inflammatory syndrome in children. J Clin Invest.

[CR30] Lei Y, Guerra Martinez C, Torres-Odio S, Bell SL, Birdwell CE, Bryant JD (2021). Elevated type I interferon responses potentiate metabolic dysfunction, inflammation, and accelerated aging in mtDNA mutator mice. Sci Adv.

[CR31] Li H, Handsaker B, Wysoker A, Fennell T, Ruan J, Homer N (2009). The sequence alignment/map format and SAMtools. Bioinformatics.

[CR32] Lima-Setta F, Magalhães-Barbosa MC, Rodrigues-Santos G, Figueiredo EADN, Jacques ML, Zeitel RS (2021). Multisystem inflammatory syndrome in children (MIS-C) during SARS-CoV-2 pandemic in Brazil: a multicenter, prospective cohort study. J Pediatr.

[CR33] Liu W, Xie Y, Ma J, Luo X, Nie P, Zuo Z (2015). IBS: an illustrator for the presentation and visualization of biological sequences. Bioinformatics.

[CR34] Marsh RA, Madden L, Kitchen BJ, Mody R, McClimon B, Jordan MB (2010). XIAP deficiency: a unique primary immunodeficiency best classified as X-linked familial hemophagocytic lymphohistiocytosis and not as X-linked lymphoproliferative disease. Blood.

[CR35] Mazer MB, Bulut Y, Brodsky NN, Lam FW, Sturgill JL, Miles SM (2022). Multisystem inflammatory syndrome in children: host immunologic responses. Pediatr Crit Care Med.

[CR36] McLaren W, Gil L, Hunt SE, Riat HS, Ritchie GR, Thormann A (2016). The Ensembl variant effect predictor. Genome Biol.

[CR37] Mehta P, Fajgenbaum DC (2021). Is severe COVID-19 a cytokine storm syndrome: a hyperinflammatory debate. Curr Opin Rheumatol.

[CR38] Melo RC, Dvorak AM (2012). Lipid body-phagosome interaction in macrophages during infectious diseases: host defense or pathogen survival strategy?. PLoS Pathog.

[CR39] Nakra NA, Blumberg DA, Herrera-Guerra A, Lakshminrusimha S (2020). Multi-system inflammatory syndrome in children (MIS-C) following SARS-CoV-2 infection: review of clinical presentation, hypothetical pathogenesis, and proposed management. Children (basel).

[CR40] Noval Rivas M, Porritt RA, Cheng MH, Bahar I, Arditi M (2021). COVID-19-associated multisystem inflammatory syndrome in children (MIS-C): a novel disease that mimics toxic shock syndrome-the superantigen hypothesis. J Allergy Clin Immunol.

[CR41] Odobasic D, Kitching AR, Holdsworth SR (2016). Neutrophil-mediated regulation of innate and adaptive immunity: the role of myeloperoxidase. J Immunol Res.

[CR42] Parodi E, Carpino A, Franchitti E, Pruccoli G, Denina M, Pagliero F (2021). Detection of faecal SARS-CoV-2 RNA in a prospective cohort of children with multisystem inflammatory syndrome (MIS-C). Epidemiol Prev.

[CR43] Porritt RA, Paschold L, Rivas MN, Cheng MH, Yonker LM, Chandnani H (2021). HLA class I-associated expansion of TRBV11-2 T cells in multisystem inflammatory syndrome in children. J Clin Invest.

[CR500] Porritt RA, Binek A, Paschold L, Rivas MN, McArdle A, Yonker LM, et al. The autoimmune signature of hyperinflammatory multisystem inflammatory syndrome in children. J Clin Invest. 2021b;131:e151520. 10.1172/JCI151520 PMC8516454 34437303

[CR44] Ramaswamy A, Brodsky NN, Sumida TS, Comi M, Asashima H, Hoehn KB (2021). Immune dysregulation and autoreactivity correlate with disease severity in SARS-CoV-2-associated multisystem inflammatory syndrome in children. Immunity.

[CR45] Reis ES, Mastellos DC, Hajishengallis G, Lambris JD (2019). New insights into the immune functions of complement. Nat Rev Immunol.

[CR46] Richards S, Aziz N, Bale S, Bick D, Das S, Gastier-Foster J (2015). Standards and guidelines for the interpretation of sequence variants: a joint consensus recommendation of the American College of Medical Genetics and Genomics and the Association for Molecular Pathology. Genet Med.

[CR47] Riphagen S, Gomez X, Gonzalez-Martinez C, Wilkinson N, Theocharis P (2020). Hyperinflammatory shock in children during COVID-19 pandemic. Lancet.

[CR49] Sacco K, Castagnoli R, Vakkilainen S, Liu C, Delmonte OM, Oguz C (2022). Immunopathological signatures in multisystem inflammatory syndrome in children and pediatric COVID-19. Nat Med.

[CR50] Sancho-Shimizu V, Brodin P, Cobat A, Biggs CM, Toubiana J, Lucas CL (2021). SARS-CoV-2-related MIS-C: a key to the viral and genetic causes of Kawasaki disease?. J Exp Med.

[CR51] Shannon P, Markiel A, Ozier O, Baliga NS, Wang JT, Ramage D (2003). Cytoscape: a software environment for integrated models of biomolecular interaction networks. Genome Res.

[CR53] Stokes EK, Zambrano LD, Anderson KN, Marder EP, Raz KM, El Burai FS (2020). Coronavirus Disease 2019 case surveillance—United States, January 22–May 30, 2020. MMWR Morb Mortal Wkly Rep.

[CR54] Strzepa A, Pritchard KA, Dittel BN (2017). Myeloperoxidase: a new player in autoimmunity. Cell Immunol.

[CR55] Syrimi E, Fennell E, Richter A, Vrljicak P, Stark R, Ott S (2021). The immune landscape of SARS-CoV-2-associated Multisystem Inflammatory Syndrome in Children (MIS-C) from acute disease to recovery. iScience..

[CR56] Takahashi T, Ellingson MK, Wong P, Israelow B, Lucas C, Klein J (2020). Sex differences in immune responses that underlie COVID-19 disease outcomes. Nature.

[CR57] Tang S, Wang J, Lee NC, Milone M, Halberg MC, Schmitt ES (2011). Mitochondrial DNA polymerase gamma mutations: an ever expanding molecular and clinical spectrum. J Med Genet.

[CR58] Vagrecha A, Zhang M, Acharya S, Lozinsky S, Singer A, Levine C (2022). Hemophagocytic lymphohistiocytosis gene variants in multisystem inflammatory syndrome in children. Biology (basel).

[CR59] Valentini P, Sodero G, Buonsenso D (2021). The relationship between COVID-19 and innate immunity in children: a review. Children (basel).

[CR60] Verdoni L, Mazza A, Gervasoni A, Martelli L, Ruggeri M, Ciuffreda M, Bonanomi E, D'Antiga L (2020). An outbreak of severe Kawasaki-like disease at the Italian epicentre of the SARS-CoV-2 epidemic: an observational cohort study. Lancet.

[CR61] Vianna EQ, Piergiorge RM, Gonçalves AP, Dos Santos JM, Calassara V, Rosenberg C (2020). Understanding the landscape of X-linked variants causing intellectual disability in females through extreme X chromosome inactivation skewing. Mol Neurobiol.

[CR62] Warren RL, Choe G, Freeman DJ, Castellarin M, Munro S, Moore R, Holt RA (2012). Derivation of HLA types from shotgun sequence datasets. Genome Med.

[CR63] Warren RL, Holt RA (2011). Targeted assembly of short sequence reads. PLoS ONE.

[CR64] Würzner R, Hobart MJ, Fernie BA, Mewar D, Potter PC, Orren A, Lachmann PJ (1995). Molecular basis of subtotal complement C6 deficiency. A carboxy-terminally truncated but functionally active C6. J Clin Invest.

[CR66] Zhang X, Shephard F, Kim HB, Palmer IR, McHarg S, Fowler GJ (2010). TILRR, a novel IL-1RI co-receptor, potentiates MyD88 recruitment to control Ras-dependent amplification of NF-kappaB. J Biol Chem.

[CR65] Zhang AQ, Liu YX, Jin JY, Wang CY, Fan LL, Xu DB (2021). Identification of a novel mutation in the C6 gene of a Han Chinese C6SD child with meningococcal disease. Exp Ther Med.

[CR67] Zhou H, Li J, Su H, Li J, Lydic TA, Young ME, Chen W (2022). BSCL2/Seipin deficiency in hearts causes cardiac energy deficit and dysfunction via inducing excessive lipid catabolism. Clin Transl Med.

[CR68] 1000 Genomes, 2022, https://www.internationalgenome.org/

[CR69] Brazilian genomic variants—AbraOM, 2022, https://abraom.ib.usp.br/

[CR70] Brazilian Initiative on Precision Medicine—BIPMed, 2022, https://bipmed.org/10.1038/s41525-020-00149-6 PMC7532430 33083011

[CR71] ClinVar, 2022, https://www.ncbi.nlm.nih.gov/clinvar/

[CR72] EnrichR, 2022, https://maayanlab.cloud/Enrichr/

[CR73] ExAC Browser, 2022, http://exac.broadinstitute.org/

[CR74] Exome Variant Server—ESP6500, 2022, https://evs.gs.washington.edu/EVS/

[CR75] Human Base, 2022, https://hb.flatironinstitute.org/

[CR76] Online Mendelian Inheritance in Man—OMIM, 2022, http://www.omim.org/17642958

[CR77] Orphanet, 2022, https://www.orpha.net/consor/cgi-bin/index.php

[CR78] Pfam platform, 2022, http://pfam.sanger.ac.uk/

[CR79] The Genome Aggregation Database—GnomAD, 2022, https://gnomad.broadinstitute.org/

[CR80] The Human Protein Atlas, 2022, https://www.proteinatlas.org/

[CR81] Variant Effect Predictor, Ensembl Genome Browser, 2022, https://www.ensembl.org/info/docs/tools/vep/index.html

[CR82] Varsome, 2022, https://varsome.com/

[CR83] WHO, World Health Organization. Multisystem inflammatory syndrome in children and adolescents with COVID-19: Scientific Brief. 2020. https://www.who.int/publications-detail/multisystem-inflammatory-syndrome-in-children-and-adolescents-with-covid-19. Accessed 27 May 27 2022.

