# Supplementary material for: Host genetic susceptibility underlying SARS-CoV-2-associated Multisystem Inflammatory Syndrome in Brazilian Children
Source: Mol Med. 2022 Dec 12;28:153. doi: 10.1186/s10020-022-00583-5 (PMC9742658; doi:10.1186/s10020-022-00583-5)
Supplement: Supplementary file 1 — Additional file 1: Table S1. Genes in which sequence variants or abnormal mRNA expression were previously associated with MIS-C (Lee et al. 2020; Chou et al. 2021; Beckmann et al. 2021; Abolhassani et al. 2022; Vagrecha et al. 2022) and genes for which genome-wide studies found association with Kawasaki disease (Sancho-Shimizu et al. 2021). [file 10020_2022_583_MOESM1_ESM.docx]

**Table S1:** Genes in which sequence variants or abnormal mRNA expression were previously associated with MIS-C (Lee *et al*, 2020; Chou *et al*, 2020; Beckmann *et al*, 2021; Abolhassani *et al*, 2022; Vagrecha *et al*, 2022) and genes for which genome-wide studies found association with Kawasaki disease (Sancho-Shimizu *et al*, 2021).

| **Condition** | **Gene name** | **Reference** |
| --- | --- | --- |
| MIS-C | *SOCS1* | Lee *et al*, 2020 |
|  | *XIAP* | Chou *et al*, 2021 |
|  | *CYBB* |  |
|  | *IFNAR1* | Abolhassani *et al*, 2022 |
|  | *LYST* | Vagrecha *et al*, 2022 |
|  | *STXBP2* |  |
|  | *PRF1* |  |
|  | *UNC13D* |  |
|  | *AP3B1* |  |
|  | *DOCK8* |  |
|  | *TBX21* | Beckmann *et al*, 2021 |
|  | *TGFBR3* |  |
|  | *C1ORF21* |  |
|  | *S1PR5* |  |
|  | *PRF1* |  |
|  | *MYBL1* |  |
|  | *KLRD1* |  |
|  | *SH2D1B* |  |
|  | *GZMA* |  |
| Kawasaki disease | FCGR2A | Sancho-Shimizu *et al*, 2021 |
|  | CASP3 |  |
|  | TRX-CAT1-7 |  |
|  | PGBD1 |  |
|  | LOC105375012 |  |
|  | LTA |  |
|  | C6ORF10 |  |
|  | HLA-DQB1 |  |
|  | HLA-DQB2-H-DOB |  |
|  | HLA-DOB |  |
|  | BLK |  |
|  | IGHV1-69 |  |
|  | ITPKC |  |
|  | CD40 |  |
