# Supplementary material for: Host genetic susceptibility underlying SARS-CoV-2-associated Multisystem Inflammatory Syndrome in Brazilian Children
Source: Mol Med. 2022 Dec 12;28:153. doi: 10.1186/s10020-022-00583-5 (PMC9742658; doi:10.1186/s10020-022-00583-5)
Supplement: Supplementary file 2 — Additional file 2: Table S2. Overview of WES data quality. [file 10020_2022_583_MOESM2_ESM.docx]

**Table S2:** Overview of WES data quality.

| **Patient code** | **Raw reads** | **Raw data (G)** | **Effective (%)** | **Coverage** | **Q20 (%)** | **Q30 (%)** |
| --- | --- | --- | --- | --- | --- | --- |
| EXOC1 | 62377984 | 12.05 | 80.38 | 134 | 96.28 | 93.12 |
| EXOC2 | 79203435 | 15.41 | 80.51 | 222 | 96.17 | 93.07 |
| EXOC3 | 66686193 | 12.88 | 81.36 | 149 | 96.82 | 93.68 |
| EXOC4 | 40388332 | 7.86 | 83.57 | 116 | 99.26 | 95.96 |
| EXOC5 | 57141299 | 11.12 | 81.71 | 163 | 97.71 | 94.71 |
| EXOC6 | 56829394 | 11.06 | 80.86 | 155 | 98.49 | 95.29 |
| EXOC7 | 62808407 | 12.13 | 77.87 | 132 | 96.72 | 93.52 |
| EXOC10 | 65231352 | 12.60 | 79.68 | 139 | 88.66 | 86.66 |
| EXOC11 | 59921694 | 11.57 | 81.70 | 135 | 97.17 | 93.99 |
| EXOC12 | 69771169 | 13.57 | 80.80 | 202 | 97.07 | 93.67 |
| EXOC13 | 69796292 | 13.58 | 81.46 | 205 | 95.28 | 92.18 |
| EXOC14 | 59629301 | 11.60 | 82.67 | 171 | 96.58 | 93.08 |
| EXOC15 | 60270776 | 11.73 | 79.72 | 167 | 95.16 | 92.36 |
| EXOC16 | 71882936 | 13.88 | 79.73 | 155 | 94.16 | 89.16 |
| EXOC17 | 49391245 | 9.54 | 77.20 | 102 | 97.16 | 94.66 |
| EXOC18 | 56824077 | 10.97 | 81.53 | 125 | 96.45 | 93.25 |
