# Supplementary material for: Host genetic susceptibility underlying SARS-CoV-2-associated Multisystem Inflammatory Syndrome in Brazilian Children
Source: Mol Med. 2022 Dec 12;28:153. doi: 10.1186/s10020-022-00583-5 (PMC9742658; doi:10.1186/s10020-022-00583-5)
Supplement: Supplementary file 3 — Additional file 3: Table S3. Sequencing metrics to support the call quality of each potentially causative variant. [file 10020_2022_583_MOESM3_ESM.docx]

**Table S3:** Sequencing metrics to support the call quality of each potentially causative variant.

| **Patient** | **Gene (Location)** | **Variant**  **Description**  **(GRCh38/hg38)** | **DP^1^** | **Allele read counts (REF, ALT)** | **Mapping quality^2^** | **Qual^3^** |
| --- | --- | --- | --- | --- | --- | --- |
| EXOC1 | *FREM1*  (9p22.3) | Chr9:14808083G>T  NM_001379081.2  c.2945C>A:p.Ser982*  rs377212852 | 266 | 135,130 | 41.970 | 3064.600 |
| EXOC3 | *MPO*  (17q22) | Chr17:58270865T>G NM_000250.2  c.2031-2A>C  rs35897051 | 248 | 115,132 | 41.940 | 3034.600 |
|  | *POLG*  (15q26.1) | Chr15:89323423A>G NM_002693.3 c.2246T>C:p.Phe749Ser  rs202037973 | 204 | 106,98 | 41.960 | 2416.600 |
| EXOC5 | *C6*  (5p13.1) | Chr5:41176504G>T NM_000065.5  c.1138delC:p.Gln380fs  rs375762365 | 214 | 111,102 | 42.000 | 3466.600 |
| EXOC6 | *ABCA4*  (1p22.1) | Chr1:94021934A>G NM_000350.3 c.4685T>C:p.Ile1562Thr  rs1762111 | 186 | 103,83 | 41.960 | 2101.600 |
| EXOC13 | *ABCA4*  (1p22.1) | Chr1:94001992C>G NM_000350.3 c.6149G>C:p.Val2050Leu  rs41292677 | 212 | 110,102 | 41.980 | 2544.600 |
| EXOC14 | *ABCA4*  (1p22.1) | Chr1:94098885C>A NM_000350.3  c.677G>T:p.Arg226Leu  rs144310835 | 278 | 129,149 | 41.960 | 3591.600 |
| EXOC15 | *C9*  (5p13.1) | Chr5:39342112G>T NM_001737.5 c.162C>A:p.Cys54*  rs34000044 | 158 | 79,79 | 42.000 | 2005.600 |
| EXOC16 | *ABCC6*  (16p13.11) | Chr16:16154974G>A NM_001171.6 c.3940C>T:p.Arg1314Trp  rs63750759 | 326 | 150,175 | 41.980 | 4187.600 |
| EXOC18 | *BSCL2*  (11q12.3) | Chr11:62694680G>GT NM_001122955.4 c.517dupA:p.Thr173fs  rs786205071 | 159 | 82,76 | 41.950 | 2272.640 |

### 1 - Depth of coverage: filtered reads that support each of the reported alleles

### 2 - Estimation of the overall root mean square (RMS) mapping quality of reads supporting a variant call

### 3 - Phred-scaled probability that a REF/ALT polymorphism exists at this site given sequencing data
