# Supplementary material for: Host genetic susceptibility underlying SARS-CoV-2-associated Multisystem Inflammatory Syndrome in Brazilian Children
Source: Mol Med. 2022 Dec 12;28:153. doi: 10.1186/s10020-022-00583-5 (PMC9742658; doi:10.1186/s10020-022-00583-5)
Supplement: Supplementary file 4 — Additional file 4: Table S4. Additional very rare variants (MAF < 0.01) found in MIS-C patients. No relevant variants were found in patient EXOC17. [file 10020_2022_583_MOESM4_ESM.docx]

**Table S4:** Additional very rare variants (MAF<0.01) found in MIS-C patients. No relevant variants were found in patient EXOC17.

| **Patient­­** | **Gene (Location)** | **Mutation**  **type** | **Mutation**  **Description**  **(GRCh38/hg38)** | **Status** | **MAF**  **(gnomAD genomes)** | **Gene function** | **Inheritance patterns (OMIM)**  ***** | **Conclusion (ACMG parameters)**  ****** |
| --- | --- | --- | --- | --- | --- | --- | --- | --- |
| EXOC1 | *AURKC*  (19q13.43) | nonsense | Chr19:57235043C>G NM_001015878.2 c.744C>G:p.Tyr248*  rs55658999 | homozygous | 0.04% | aurora kinase C | AR | pathogenic  (PVS1, PM2, PP5) |
| EXOC2 | *FBN1*  (15q21.1) | missense | Chr15:48510133T>C NM_000138.5 c.1625A>G:p.Asn542Ser  rs963564435 | heterozygous | NA | fibrillin 1 | AD | likely pathogenic  (PM1, PP2, PM2, BP4) |
| EXOC4 | *NAXD*  (13q34) | nonsense | Chr13:110638477C>T NM_018218.4  c.1129C>T:p.Arg377*  rs114275451 | heterozygous | 0.51% | NAD(P)HX dehydratase | AR | likely pathogenic  (PVS1, PM2, BS2) |
| EXOC5 | *USH2A*  (1q41) | frameshift | Chr1:2163255552TG>T NM_206933.4 c.895delC:p.Gln299fs  rs1338169194 | heterozygous | <0.01% | usherin | AR/UN | pathogenic  (PVS1, PM2, PP5) |
| EXOC7 | *ELAC2*  (17p12) | missense | Chr17:12992957C>T NM_018127.7 c.2342G>A:p.Arg781His  rs119484086 | heterozygous | 0.05% | elaC ribonuclease Z 2 | AR/UN | VUS  (PM2, PP3, PP5) |
| EXOC10 | *ALDH7A1*  (5q23.2) | frameshift | Chr5:126595164GC>G NM_001182.5 c.34delG:p.Ala12fs  rs750693623 | heterozygous | 0.05% | aldehyde dehydrogenase 7 family member A1 | AR | likely pathogenic  (PVS1, PP5) |
| EXOC11 | *EDNRB*  (13q22.3) | nonsense | Chr13:77919522C>A  NM_001201397.1 c.94G>T:p.Gly32*  rs199556320 | heterozygous | 0.01% | endothelin receptor type B | AR/AD | likely pathogenic  (PVS1, PM2) |
|  | *OTX2*  (14q22.3) | missense | Chr14:56802204G>C  NM_021728.4 c.425C>G:p.Pro142Arg  rs199761861 | heterozygous | 0.02% | orthodenticle homeobox 2 | AD | benign  (BS1, BS2, PP2) |
| EXOC12 | *PKLR*  (1q22) | missense | Chr1:155294618C>T  NM_000298.6  c.829G>A:p.Glu277Lys  rs147689373 | heterozygous | 0.25% | pyruvate kinase L/R | AR/AD | benign  (BS1, BS2, PP2) |
| EXOC13 | *SLC39A5*  (12q13.3) | frameshift | Chr12:5623699CAGTG>C  NM_173596.3  c.945_945+3delAGTG  rs746925912 | heterozygous | <0.01% | solute carrier family 39 member 5 | AD | likely benign  (BS2) |
| EXOC15 | *EVC*  (4p16.2) | splice donor | Chr4:5809612G>T NM_153717.3  c.2782+1G>T  rs1007534611 | heterozygous | <0.01% | EvC ciliary complex subunit 1 | AR/AD | pathogenic  (PVS1, PM2, PP5) |
| EXOC16 | *EXT2*  (11p11.2) | frameshift | Chr11:44236296AC>A NM_207122.2  c.1943delC: p.Pro648fs  no rs ID | heterozygous | NA | exostosin glycosyltransferase 2 | AR/AD | pathogenic  (PVS1, PM2) |
|  | *GNAT1*  (3p21.31) | nonsense | Chr3:50193573C>A NM_144499.3 c.359C>A:p.Ser120*  rs778497200 | heterozygous | <0.01% | G protein subunit alpha transducin 1 | AR/AD | pathogenic  (PVS1, PM2, PP5) |
| EXOC18 | *CAPN3*  (15q15.1) | missense | Chr15:42409982T>C NM_000070.3 c.2102T>C:p.Ile701Thr  rs1008776680 | heterozygous | <0.01% | calpain 3 | AR/AD | likely pathogenic  (PM2, BS2, PM1, PP2, PP3) |

### VUS: variant of unknown significance; NA: not available; * According to OMIM - Online Mendelian Inheritance in Man®; ** According to ACMG guidelines (Richards *et al*, 2015) and Varsome.
