# Supplementary material for: Host genetic susceptibility underlying SARS-CoV-2-associated Multisystem Inflammatory Syndrome in Brazilian Children
Source: Mol Med. 2022 Dec 12;28:153. doi: 10.1186/s10020-022-00583-5 (PMC9742658; doi:10.1186/s10020-022-00583-5)
Supplement: Supplementary file 6 — Additional file 6: Table S6. Previous host genetic variants described in patients with MIS-C by whole exome sequencing or immune-gene-panel. [file 10020_2022_583_MOESM6_ESM.docx]

**Table S6:** Previous host genetic variants described in patients with MIS-C by whole exome sequencing or immune-gene-panel.

| **Gene** | **Variant** | **Status** | **Mutation type** | **Sex, age** | **Gene function** | **Methodology** | **Reference** |
| --- | --- | --- | --- | --- | --- | --- | --- |
| *SOCS1* | NM_003745.1:c.24delA  p.Ala9Profs*76 | heterozygous | frameshift | male, 17 ys | negative regulator of type I and II IFN signalling | WES | Case report; Lee *et al* (2020) |
| *XIAP* | [NM_001167.4](https://www.ncbi.nlm.nih.gov/nuccore/NM_001167.4):c.1262G>A  p.Ser421Asn | hemizygous | missense | male, 11 ys | cellular survival, activation, and negative regulation of NLRP3 inflammasome | WES | MIS-C cohort (n=18); Chou *et al* (2021) |
| *CYBB* | NM_000397.4^#^  p.Arg229Thr | hemizygous | missense | male, 16 ys | *CYBB* encodes the p91^phox^ subunit of the NADPH oxidase | WES | MIS-C cohort (n=18); Chou *et al* (2021) |
| *IFNAR1* | NM_000629^#^  p.H263fs14* | homozygous | large frameshift deletion (4.394 bp) | female, 3 ys | IFNAR1 protein belongs to the type II cytokine receptor family and functions as an antiviral factor | WES | Case report with concomitant critical COVID-19 pneumonia and MIS-C; Abolhassani *et al* (2022) |
| *LYST* | NM_001301365.1:c.2030C>T  p.Ile677Thr | heterozygous | missense | female, 8 ys | lysosomal trafficking regulator | 109-immune-gene-panel | MIS-C cohort (n=39); Vagrecha *et al* (2022) |
| *LYST* | NM_001301365.1:c.10669G>T  p.Val3557Leu | heterozygous | missense | male, 7 ys | lysosomal trafficking regulator |  |  |
| *STXBP2* | NM_001127396.3:c.1772C>A  p.Ala591Asp | heterozygous | missense | female, 3 ys | syntaxin binding protein 2 |  |  |
| *PRF1* | NM_001083116.3:c.1424G>A  p.Gly475Glu | heterozygous | missense | female, 14 ys | perforin 1 |  |  |
| *UNC13D* | NM_199242.3:c.796C>T  p.Arg266Cys | heterozygous | missense | male, 8 ys | unc-13 homolog D |  |  |
| *AP3B1* | NM_001271769.2:c.1862C>G  p.Thr621Ser | heterozygous | missense | female, 14 ys | adaptor related protein complex 3 subunit beta 1 |  |  |
| *DOCK8* | NM_001193536.2:c.40G>A  p.Ala2Thr | heterozygous | missense |  | dedicator of cytokinesis 8 |  |  |
| *DOCK8* | NM_001193536.2:c.2060C>T  p.Pro687Leu | heterozygous | missense | male, 10 ys | dedicator of cytokinesis 8 |  |  |
| *DOCK8* | NM_001193536.2:c.2695C>T  p.Arg899Trp | heterozygous | missense | male, 4 ys | dedicator of cytokinesis 8 |  |  |
| *DOCK8* | NM_001193536.2:c.1193G>A  p.Arg398Gln | heterozygous | missense | male, 2 ys | dedicator of cytokinesis 8 |  |  |

^#^ The position of the variant was not described at cDNA level by the authors.
