# Supplementary material for: Host genetic susceptibility underlying SARS-CoV-2-associated Multisystem Inflammatory Syndrome in Brazilian Children
Source: Mol Med. 2022 Dec 12;28:153. doi: 10.1186/s10020-022-00583-5 (PMC9742658; doi:10.1186/s10020-022-00583-5)
Supplement: Supplementary file 7 — Additional file 7: Figure S1. Workflow of the methodology used. Figure S2. Electropherograms obtained by Sanger sequencing, confirming the WES results. Figure S3. Location of the potentially causative variants in the canonical protein forms obtained from the UniProt Platform. The proteins, proportional to size, the positions of the mutations (lollipops), and the coordinates of the domains annotated in Pfam are represented. Variants identified in FREM1, C6, C9, ABCC6, and BSCL2 are contained in the functional domains of their proteins, whereas for the ABCA4 variant, two variants are outside functional domains and one is within it. For POLG, the variant is located outside functional domains. MPO variant was not illustrated, since it is intronic. Figure S4. Highly reliable protein–protein interactions network retrieved from the products of the eight genes studied. The red hexagons represent the genes carrying the variants. The circles represent the interactions obtained through the STRING plugin, with the green circles representing genes already related to the MIS-C phenotype and blue circles representing genes already related to Kawasaki disease. Figure S5. Enrichment analysis through EnrichR tool of the proteins verified in the PPIs network (FDR < 0.05) against the following gene-set libraries: KEGG 2021 (a); Gene Ontology 2021—biological processes (b), molecular function (c), and cellular component (d); Jensen tissues (e); the scRNA-seq database PanglaoDB Augmented 2021 (f); COVID-19-related gene sets 2021 (g). Top ten elements were sorted by adjusted p-value ranking. [file 10020_2022_583_MOESM7_ESM.pptx]

## Slide 1
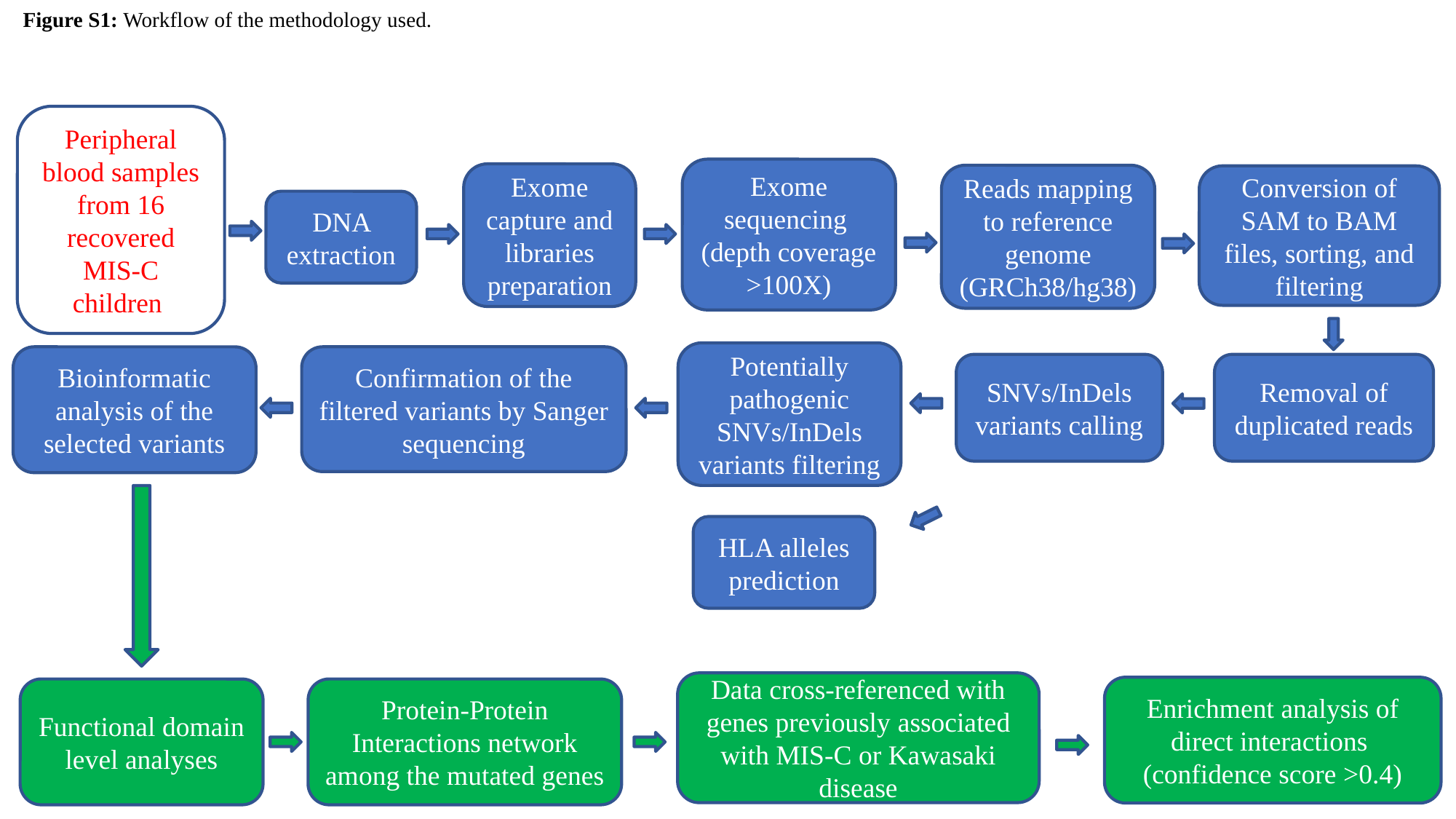

Figure S1: Workflow of the methodology used.
Peripheral blood samples from 16 recovered MIS-C children
Exome sequencing
(depth coverage >100X)
Exome capture and libraries preparation
Reads mapping to reference genome (GRCh38/hg38)
Conversion of SAM to BAM files, sorting, and filtering
DNA extraction
Potentially pathogenic SNVs/InDels variants filtering
Bioinformatic analysis of the selected variants
Confirmation of the filtered variants by Sanger sequencing
SNVs/InDels variants calling
Removal of duplicated reads
HLA alleles prediction
Data cross-referenced with genes previously associated with MIS-C or Kawasaki disease
Enrichment analysis of direct interactions (confidence score >0.4)
Functional domain level analyses
Protein-Protein Interactions network among the mutated genes

## Slide 2
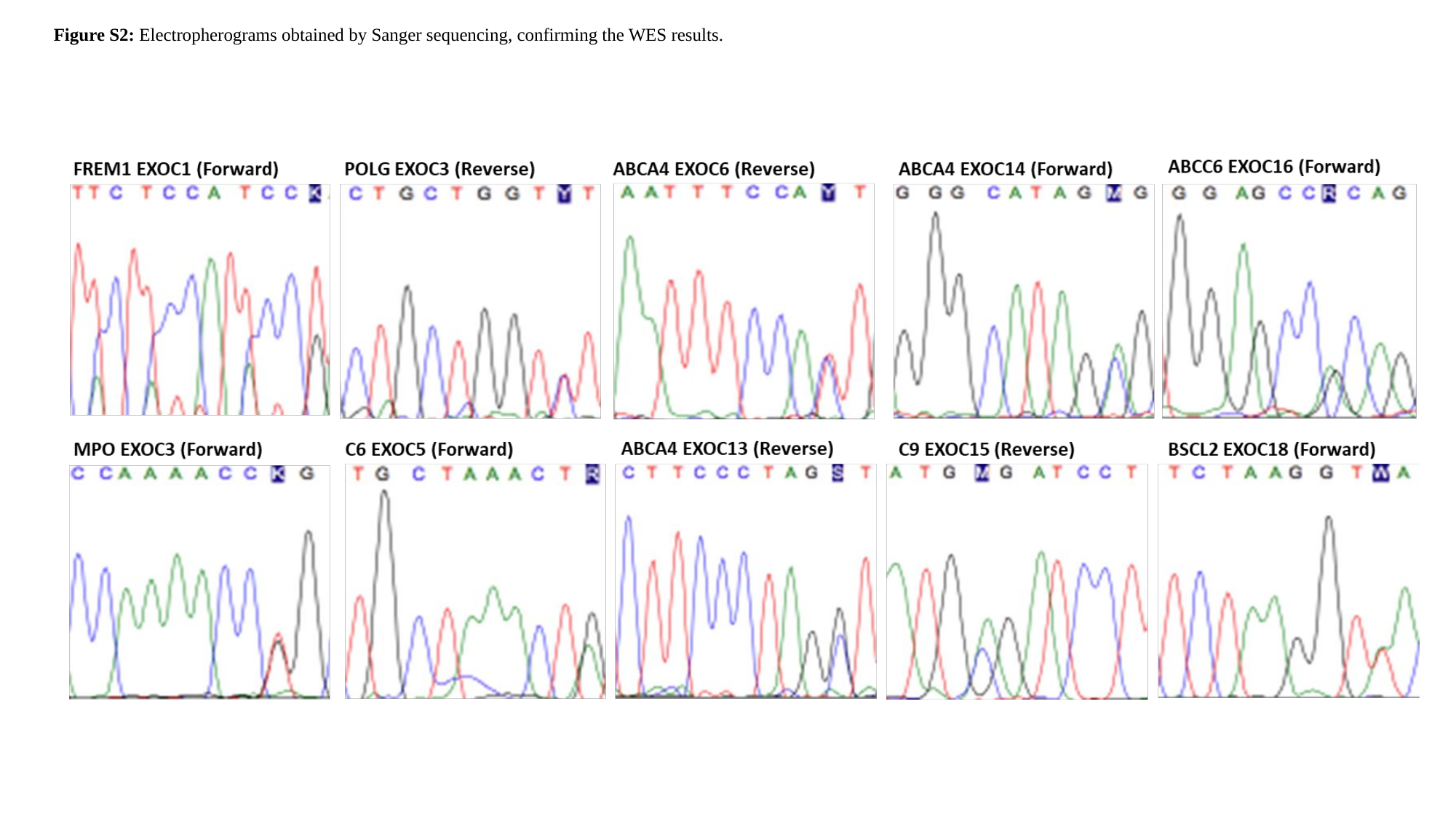

Figure S2: Electropherograms obtained by Sanger sequencing, confirming the WES results.

## Slide 3
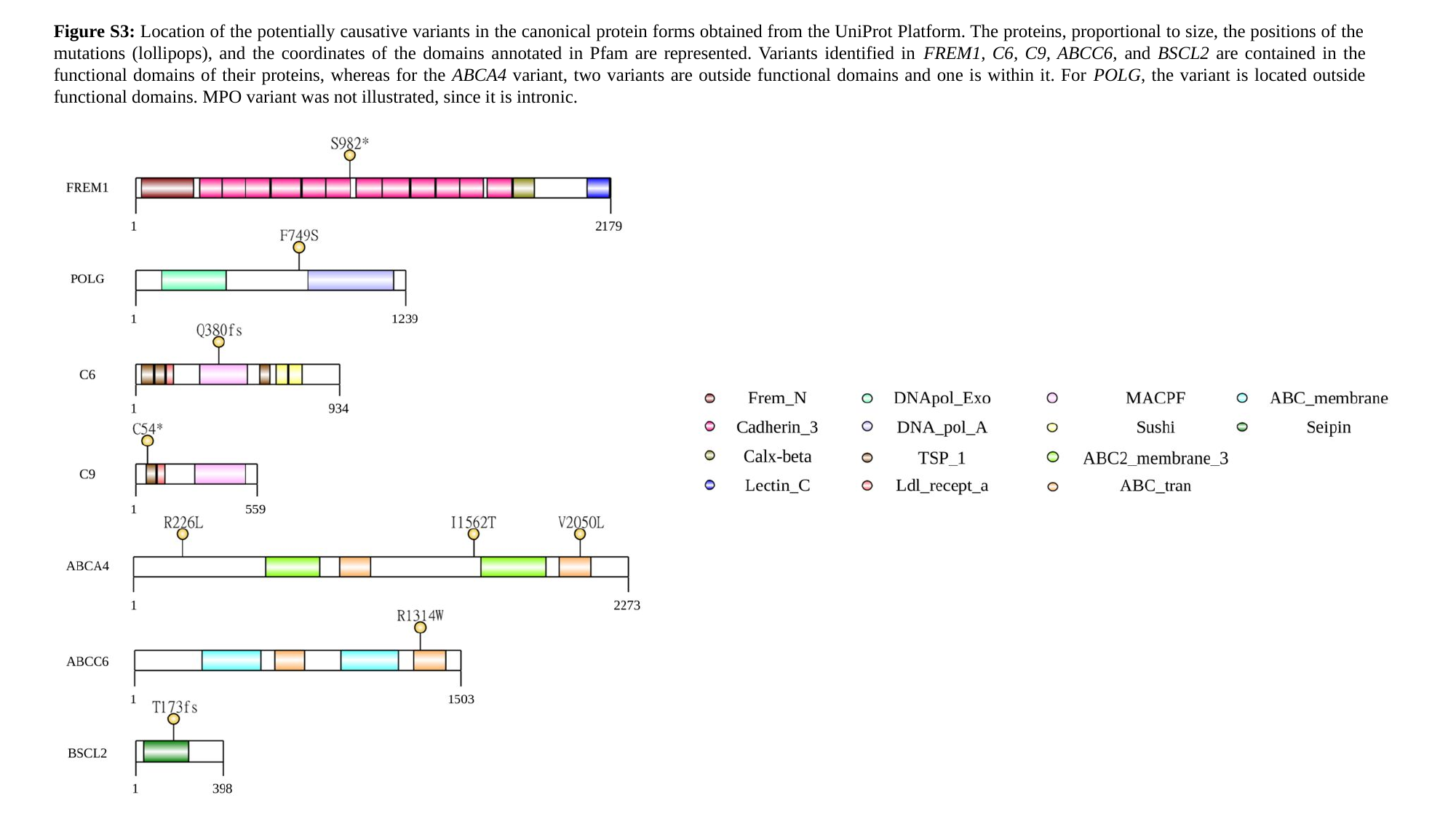

Figure S3: Location of the potentially causative variants in the canonical protein forms obtained from the UniProt Platform. The proteins, proportional to size, the positions of the mutations (lollipops), and the coordinates of the domains annotated in Pfam are represented. Variants identified in FREM1, C6, C9, ABCC6, and BSCL2 are contained in the functional domains of their proteins, whereas for the ABCA4 variant, two variants are outside functional domains and one is within it. For POLG, the variant is located outside functional domains. MPO variant was not illustrated, since it is intronic.

## Slide 4
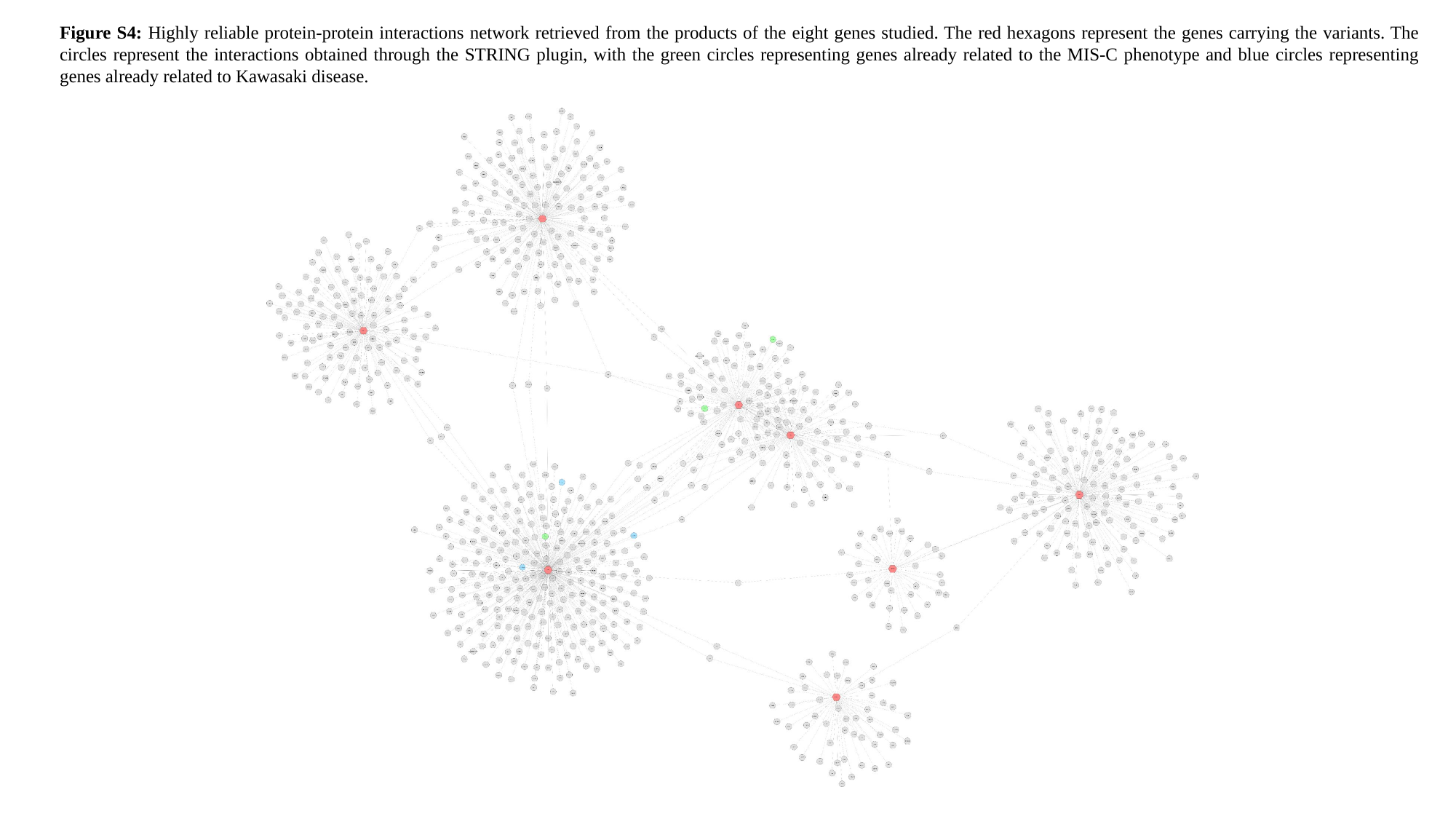

Figure S4: Highly reliable protein-protein interactions network retrieved from the products of the eight genes studied. The red hexagons represent the genes carrying the variants. The circles represent the interactions obtained through the STRING plugin, with the green circles representing genes already related to the MIS-C phenotype and blue circles representing genes already related to Kawasaki disease.

## Slide 5
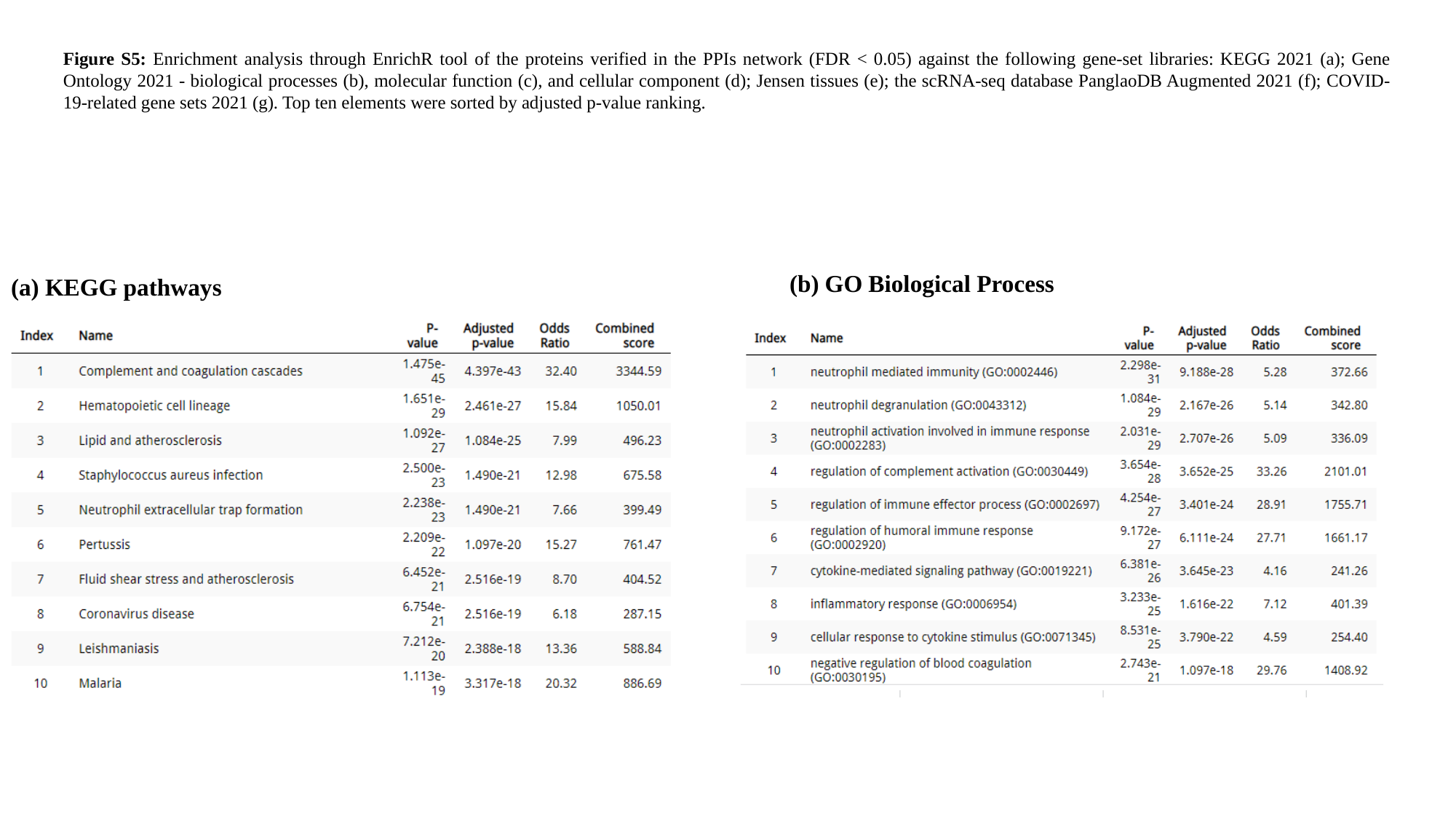

Figure S5: Enrichment analysis through EnrichR tool of the proteins verified in the PPIs network (FDR < 0.05) against the following gene-set libraries: KEGG 2021 (a); Gene Ontology 2021 - biological processes (b), molecular function (c), and cellular component (d); Jensen tissues (e); the scRNA-seq database PanglaoDB Augmented 2021 (f); COVID-19-related gene sets 2021 (g). Top ten elements were sorted by adjusted p-value ranking.
(b) GO Biological Process
(a) KEGG pathways

## Slide 6
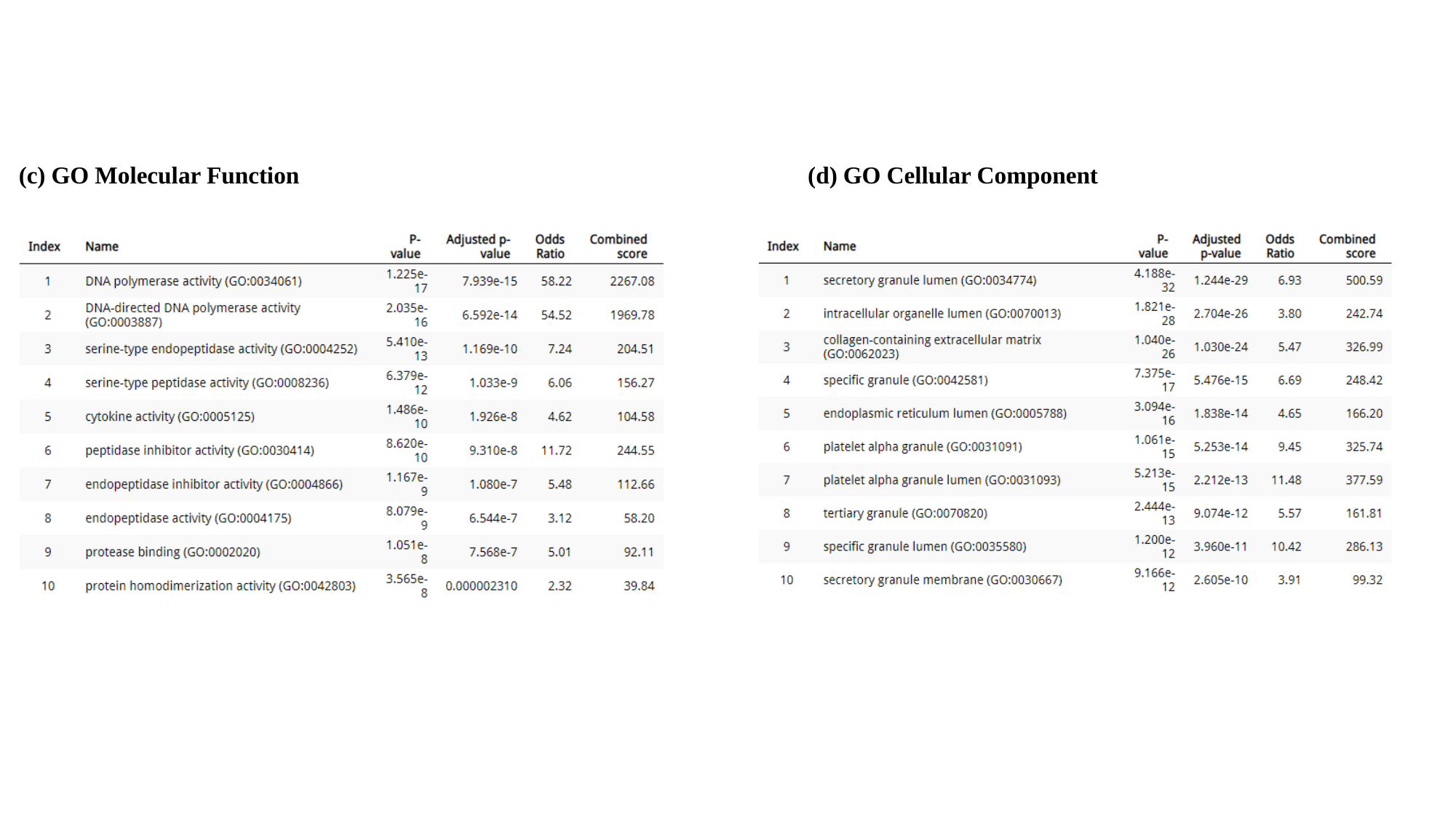

(c) GO Molecular Function
(d) GO Cellular Component

## Slide 7
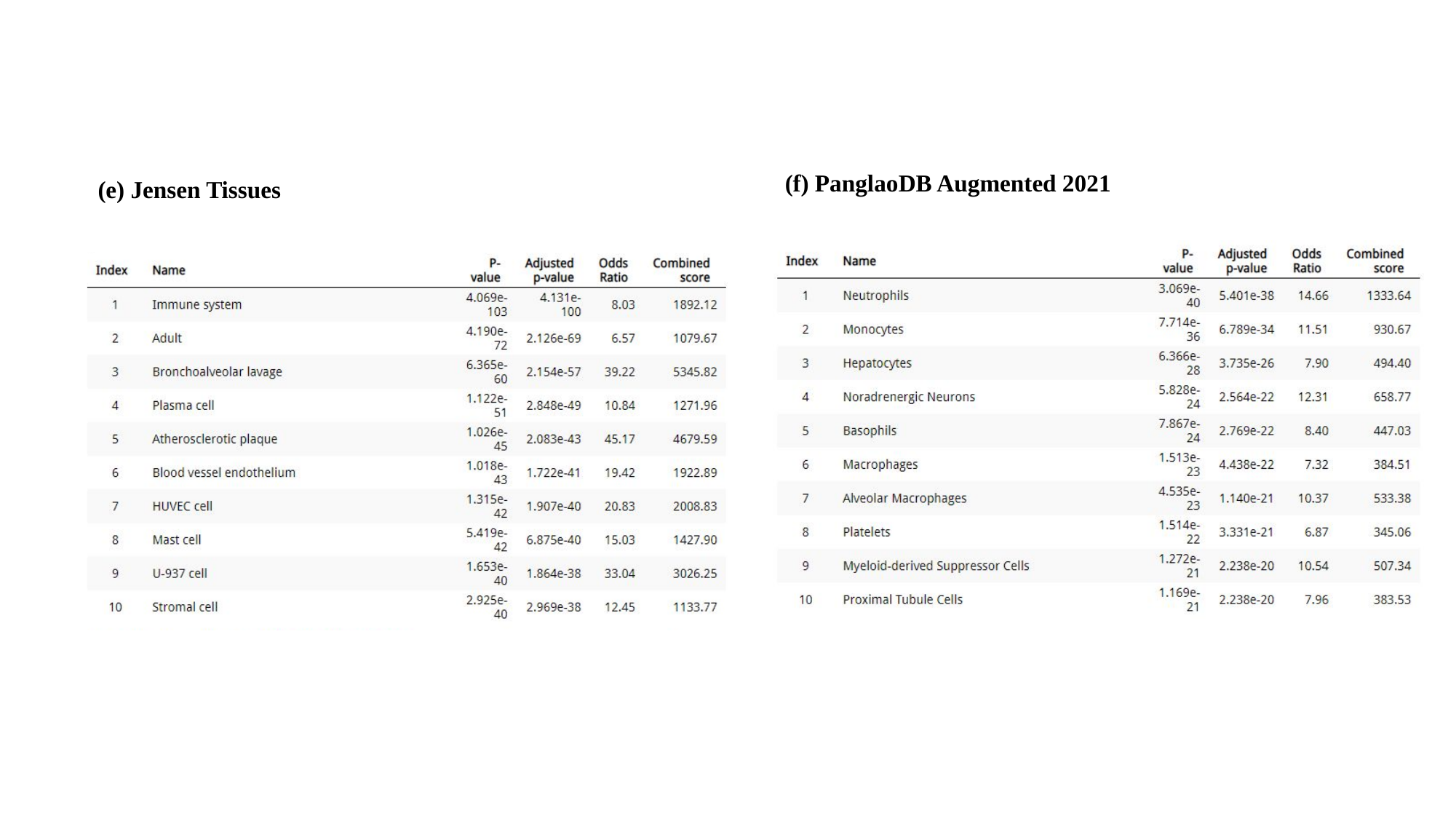

(f) PanglaoDB Augmented 2021
(e) Jensen Tissues

## Slide 8
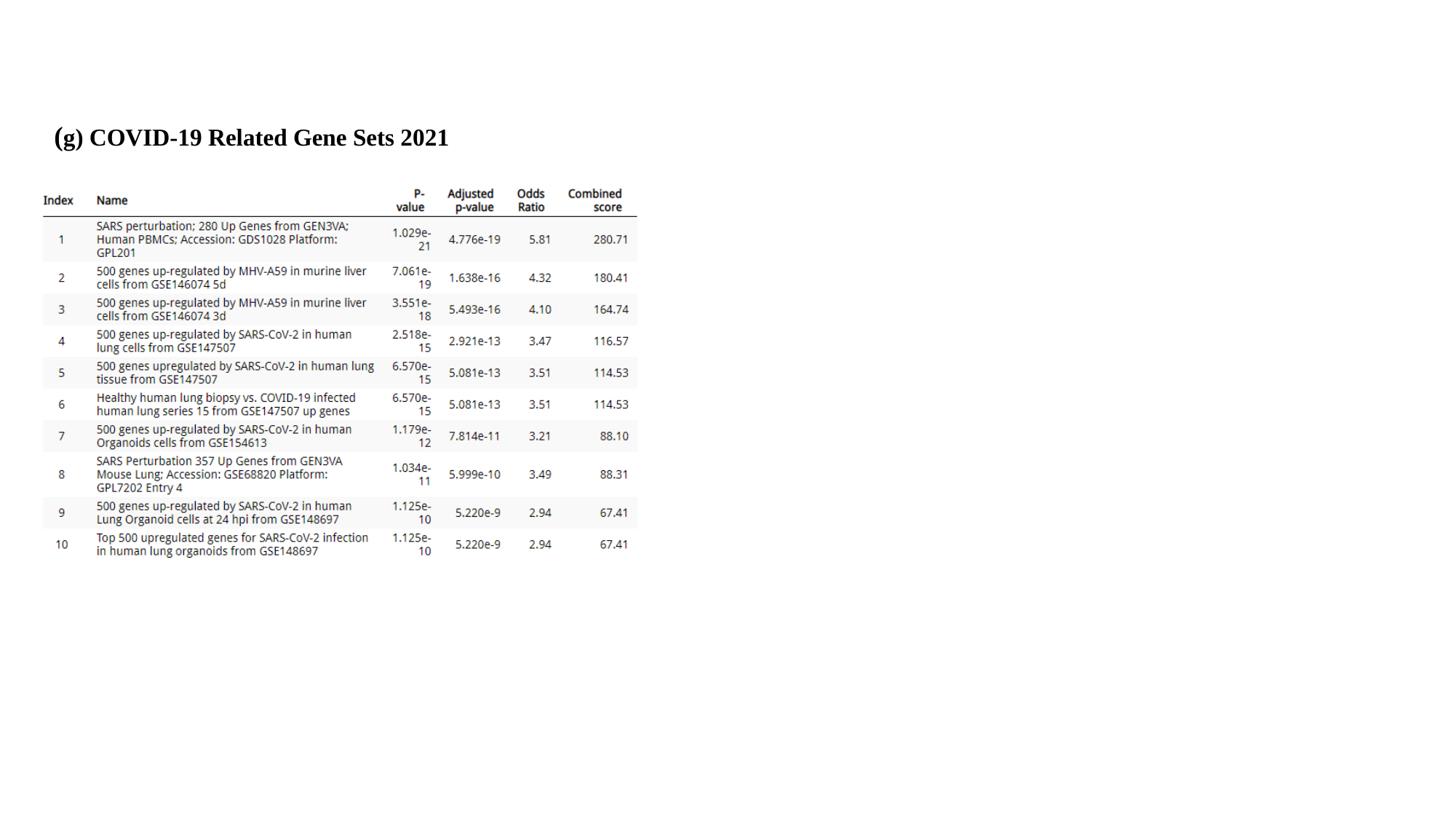

(g) COVID-19 Related Gene Sets 2021
